# Supplementary material for: The validity and reliability of observational assessment tools available to measure fundamental movement skills in school-age children: A systematic review
Source: PLoS One. 2020 Aug 25;15(8):e0237919. doi: 10.1371/journal.pone.0237919 (PMC7447071; doi:10.1371/journal.pone.0237919)
Supplement: S1 Table — (DOCX) [file pone.0237919.s002.docx]

Supporting information 1 – Search Strategy

| **Assessment tools terms** | **Reliability/ validity search terms** | **Population search terms** |
| --- | --- | --- |
| Movement Assessment Battery for Children | Valid* | Child* |
| MABC | Reliab* | Infant* |
| MABC 2 | Accura* | School child* |
| MABC 2 | Feasib* | Adolescen* |
| M-ABC | Consisten* | Preschool |
| Movement-ABC | Agreement | Pre-school |
| Movement-ABC 2 | Precision | Boy* |
| Movement-ABC-2 | Psychometric propert* | Girl* |
| Movement ABC | Repeatab* | Young people |
| Movement ABC 2 | Reproducib* | Teenager |
| Movement ABC-2 | Convergent | Youth |
| Assessment of Perceptual and  Fundamental Motor Skills  Inventory | divergent |  |
| APM Inventory |  |  |
| APM-Inventory |  |  |
| Fundamental Motor Skills Test  Package |  |  |
| FMS Test Package |  |  |
| Fundamental Movement Skill Polygon |  |  |
| FMS Polygon |  |  |
| FMS-Polygon |  |  |
| Peabody Developmental Motor Scale |  |  |
| PDMS |  |  |
| PDMS 2 |  |  |
| PDMS-2 |  |  |
| Get Skilled Get Active |  |  |
| GSGA |  |  |
| NSW Department of Education and Training resource on FMS |  |  |
| New South Wales Department of Education and Training resource on FMS |  |  |
| NSW Department of Education and Training resource on Fundamental Movement Skills |  |  |
| New South Wales Department of Education and Training resource on Fundamental Movement Skills |  |  |
| Körperkoordinationstest für Kinder |  |  |
| Korperkoordinationstest fur kinder |  |  |
| KTK |  |  |
| Test of Gross Motor Development |  |  |
| TGMD |  |  |
| TGMD 2 |  |  |
| TGMD-2 |  |  |
| TGMD 3 |  |  |
| TGMD-3 |  |  |
| Bruninks-Oseretsky Test of Motor Proficiency |  |  |
| BOTMP |  |  |
| BOTMP 2 |  |  |
| BOTMP-2 |  |  |
| BOT |  |  |
| BOT-2 |  |  |
| BOT 2 |  |  |
| Furtado-Gallagher Computerized Observational Movement Pattern Assessment System |  |  |
| FG-COMPASS |  |  |
| Motoriktest für vier-bis sechsjährige Kinder |  |  |
| Motoriktest fur vier-bis sechsjahrige Kinder |  |  |
| MOT 4-6 |  |  |
| Ohio State University Scale of Intra Gross Motor Assessment |  |  |
| OSU-SIGMA |  |  |
| SIGMA |  |  |
| Athletic Skills Track |  |  |
| AST |  |  |
| Canadian Agility and Movement Skill Assessment |  |  |
| CAMSA |  |  |
| Children's Activity and Movement in Preschool Motor Skills Protocol |  |  |
| CMSP |  |  |
| CHAMPS |  |  |
| CHAMPS motor skills protocol |  |  |
| Early years movement skills checklist |  |  |
| EYMSC |  |  |
| Fundamental Motor Skill Stage Characteristics |  |  |
| Fundamental Movement Screen |  |  |
| Fundamental Movement Screen Test |  |  |
| Fundamental movement skill assessment tool |  |  |
| Instrument for the Evaluation of Fundamental Movement Patterns |  |  |
| Instrumento de Evaluacion  de los Patrones Basicos de Movimiento |  |  |
| IPBM |  |  |
| Lifelong Physical Activity Skills Battery |  |  |
| NSW Schools Physical Activity and Nutrition Survey |  |  |
| New South Wales Schools Physical Activity and Nutrition Survey |  |  |
| NSW SPANS |  |  |
| New South Wales SPANS |  |  |
| Objectives-Based Motor Skill Assessment Instrument |  |  |
| Passport for Life |  |  |
| PE Metrics |  |  |
| PLAYbasic |  |  |
| PLAYfun |  |  |
| Preschooler gross motor quality scale |  |  |
| PGMQ |  |  |
| Smart Start |  |  |
| Smart start-2 |  |  |
| Smart Start 2 |  |  |
| Teen Risk Screen |  |  |
| Test for FMS in Adults |  |  |
| Test for Fundamental Movement Skills in Adults |  |  |
| TFMSA |  |  |
| Instrumento para la evaluacion de Patrones Basicos de Movimiento |  |  |
| IPBM |  |  |
| Victorian FMS Assessment instrument |  |  |
| Victorian Fundamental Movement Skills Assessment Instrument |  |  |
| Victorian FMS Teacher* Manual Assessment |  |  |
| Victorian Fundamental Movement Skill Teacher* Manual Assessment |  |  |
| Victorian Fundamental Motor Skills manual |  |  |
| Department of Education of Victoria A Fundamental Motor Skills: A Manual for Classroom Teachers |  |  |
| Western Australian Stay in Step Screening Assessment |  |  |
| Western Australian Department of Education Steps Resource: The Stay in Step Screening Assessment |  |  |
| Stay in step |  |  |
