# Supplementary material for: The validity and reliability of observational assessment tools available to measure fundamental movement skills in school-age children: A systematic review
Source: PLoS One. 2020 Aug 25;15(8):e0237919. doi: 10.1371/journal.pone.0237919 (PMC7447071; doi:10.1371/journal.pone.0237919)
Supplement: S2 Table — (DOCX) [file pone.0237919.s003.docx]

Supporting information 2 – study table

| **Citation** | **Assessment tool(s) used** | **Sample** | **Outcome** | **Statistics used** | **Results** | **Methodology quality rating (%)** |
| --- | --- | --- | --- | --- | --- | --- |
| Africa & Kidd (2013) | Teen risk screen | Girls = 125  (mean age = 12.12; SD= 1.1)  Teachers = 7 | Internal Consistency | Cronbach’s alpha | Posture and stability (axial movement) time 1 = .93, time 2 = .86    Posture and stability (dynamic movement) time 1 = .89, time 2 = .86    Locomotor (single skills) time 1 = .89, time 2 = .90    Locomotor (combination) time 1= .67, time 2 = .56    Manipulative Skills (sending away) time 1 = .71, time 2 = .45    Manipulative skills (maintaining possession) = .58 | **36%** |
|  |  |  | Test-retest reliability | Pearson correlation | Posture and stability (axial movement) = .59  Posture and stability (dynamic movement) = .69    Locomotor (single skills) = .88    Locomotor (combination) = .76    Manipulative Skills (sending away) = .43    Manipulative skills (maintaining possession) = .58 |  |
|  |  |  |  | Intraclass correlation | Posture and stability (axial movement) = .51 (.32, .65)    Posture and stability (dynamic movement) = .63 (.46, .75)    Locomotor (single skills) = .86 (.76, .91)    Locomotor (combination) = .74 (.65, .82)    Manipulative Skills (sending away) = .34 (.13, .51)    Manipulative skills (maintaining possession) = .56 (.42, .67) |  |
|  |  |  | Structural validity | Confirmatory Factor analysis | Postural stability (axial movement) and postural stability (dynamic movement) test 1 scales - marginal fit statistics with the RMSEA just outside the prescribed boundaries (<.05). AGFA indices were acceptable (>.95).    Test 2 fit statistics were well below acceptable. Locomotor (single skills) - marginally acceptable RMSEA (Test 1 and 2) and acceptable AGFI, CR and VE (Test 1 and 2).    3-scale CFA model gave acceptable results for all indices at both time points with perhaps the VE of manipulative skills (sending away) at time Test 2 being slightly lower (VE=0.43). |  |
| Allen et al. (2017) | TGMD-3 | 14 children with ASD (age range 4-10)    21 typically developing children, aged 4 - 11. | Internal consistency | Cronbach’s alpha | TGMD-3  Typically developing group:  Locomotor skills= 0.70; Ball Skills= 0.6; Overall= 0.74  ASD- traditional protocol:  Locomotor skills= 0.82; Ball Skills= 0.75; Overall= 0.88  ASD- visual protocol:  Locomotor skills= 0.93; Ball skills= 0.81; Overall= 0.93  TGMD-2  Locomotor= 0.85; Ball skills= 0.88; Overall= 0.91 | **55%** |
|  |  |  | Inter-rater reliability | Intra-class correlational coefficient with 95% confidence limits | ASD visual:  Locomotor= 0.98 (0.94, 1.00)  Ball skills= 0.96 (0.86, 0.99)  Overall= 0.99 (.95, 1.00)  ASD traditional:  Locomotor= 0.98 (0.92, 0.99)  Ball skills= 0.97 (0.91, 0.99)  Overall= 0.98 (0.94, 1.00)  Typically developing:  Locomotor= 0.91 (0.79, 0.96)  Ball skills= 0.92 (0.81, 0.97)  Overall= 0.94 (0.87, 0.98) |  |
|  |  |  | Intra-rater reliability |  | ASD visual:  Locomotor= 0.99 (.95, 1.00)  Ball skills= 1.00 (0.98, 1.00)  Overall= 0.99 (0.98, 1.00)  ASD traditional:  Locomotor= 0.97 (0.88, 0.99)  Ball skills= 0.99 (0.96, 1.00)  Overall= 0.99 (0.92, 1.00)  Typically developing:  Locomotor= 0.97 (0.93, 0.99)  Ball skills= 0.91 (0.68, 0.97)  Overall= 0.95 (0.84, 0.98) |  |
|  |  |  | Test-retest reliability |  | ASD visual:  Locomotor= 0.92 (0.65, 0.98)  Ball skills= 0.83 (0.39, 0.96)  Overall= 0.92 (0.66, 0.98)  ASD traditional:  Locomotor= 0.92 (0.65, 0.98)  Ball skills= 0.82 (0.31, 0.96)  Overall= 0.91 (0.63, 0.98)  Typically developing:  Locomotor= 0.81 (0.94, 0.53)  Ball skills= 0.84 (0.62, 0.94)  Overall= 0.92 (0.78, 0.97) |  |
|  |  |  | Structural validity | Two-tailed independent t-test | Differences between TGMD-3 traditional protocol (typically developing and ASD):  Locomotor - *t*=3.75, *p*=.001  Ball Skills - *t*=3.51, *p*=.002  Overall - *t*=3.93, *p*=.001  typically developing group scored significantly higher on the TGMD-3 traditional protocol than the ASD group  Positive correlations between TGMD-3 traditional protocol and the TGMD-3 visual protocol scores:  Locomotor - *r*(10)=0.94, *p*<0.001, 95% CI (0.80, 0.98)  Ball Skills - *r*(10)=0.93, *p*<0.001, 95% CI (0.76, 0.98)  Overall - *r*(10)=0.96, *p* < 0.001, 95% CI (0.86, 0.99). |  |
| Bakke et al (2017) | MABC- 2 Portuguese version | Children= 30 (17 boys; 13 girls; mean age = 9.44; SD= 1.08)  Moderate low vision= 28  Severe low vision= 2 | Test-retest reliability | Intraclass correlational coefficient with 95% confidence limits | A1xA2 (rater 1 in the first application; rater 1 in the second application)  Aiming and catching= 0.867 (0.676, 0.945)  Balancing = 0.856 (0.651, 0.941)  Total= 0.958 (0.899, 0.983)  B1xB2 (rater 2 in the first application; rater 2 in the second application)  Aiming and catching= 0.847 (0.628, 0.936)  Balancing= 0.834 (0.596, 0.931) | **50%** |
|  |  |  | Inter-rater reliability | Intraclass correlational coefficient with 95% confidence limits | A1xB2 (rater 1 in the first application; rater 2 in the first application)  Aiming and catching= 0.957 (0.905, 0.980)  Balance= 0.936 (0.867, 0.970)  Total= 0.971 (0.939, 0.986) |  |
|  |  |  | Internal consistency | Cronbach’s alpha | Cronbach’s alpha ranged from 0.790 to 0.868. |  |
| Bardid et al (2016) | KTK  MOT 4-6 | 638 young children (323 boys; 315 girls)  aged 5-6. | Concurrent Validity  Hypothesis Testing Validity | Spearman's rank | KTK MQ and MOT 4-6 MQ (*r*s = .63)  KTK MQ and MOT 4-6 gross motor cluster score (*r*s = .62)  KTK MQ and MOT 4-6 locomotor score (*r*s = .56)  KTK MQ and MOT 4-6 stability score (*r*s = .43)  KTK MQ and MOT 4-6 object-control score (*r*s = .37).  KTK MQ and MOT 4-6 ne motor cluster score (*r*s = .32).  MQs of both tests (*r*s = .61–.67)  KTK MQ and MOT 4-6 gross motor score (*r*s = .62–.72).  KTK MQ and MOT 4-6 locomotor score (*r*s = .53–.68)  KTK MQ and MOT 4-6 stability score (*r*s = .42–.49)  KTK MQ and MOT 4-6 object-control score (*r*s = .31–.44)  KTK MQ and MOT 4-6 ne motor cluster score (*r*s = .20–.47). | **47%** |
|  |  |  |  | Cohen’s Kappa | KTK and MOT 4-6 at P2 (.50)  P16 (.52),    P84 (.23),  P98 (.00). |  |
| Bardid et al. 2016b | TGMD-2 | 1614 children aged 3-8 years  841 boys, 773 girls | Cross cultural validity | Chi-squared | Belgian children performed significantly worse than US norm sample on GMQ (chi-square = 219.548, p < 0.001, Cramer’s V = 0.279).  Belgian children’s performed worse on the locomotor  (chi-square = 147.872, p < 0.001, Cramer’s V = 0.229) and object control subtests (chi-square = 357.94, p < 0.001, Cramer’s V = 0.356) | **60%** |
|  |  |  | Hypotheses testing validity | T-tests | No significant differences between Belgian and US boys on the locomotor subtest in the age groups of three (t = 0.961, p = 0.338), four (t= 1.735, p = 0.084) and five (t = 1.300, p = 0.195)  No significant difference between Belgian and US three year-old girls (t = -0.828, p = 0.410) and four-year-old girls (t = 1.233, p = 0.220),  Five year-old Belgian girls scored significantly higher on locomotor skills (t = 4.813, p < 0.001, Cohen’s d = 0.4).  Lower locomotor skill performances for Belgian boys and girls aged six years (boys t = -5,632, p <  0.001, Cohen’s d = 0.446 and girls t = -2.193, p = 0.030, Cohen’s d = 0.161), seven years (boys  t = -4.036, p < 0.001, Cohen’s d = 0.396 and girls t = -3.106, p = 0.002, Cohen’s d = 0.306) and  eight years (boys t = -3.577, p = 0.001, Cohen’s d = 0.453 and girls t = -9.717, p < 0.001,  Cohen’s d = 1.095)  Belgian children of all age groups performed significantly worse on object control skills than the US reference population (all p-values < 0.001, Cohen’s d = 0.303-1.269). |  |
| Bardid, Utesch, & Lenoir (2019) | BOT-2 SF | 2538 children aged 6‐11 years from urban and rural areas. | Structural Validity | Rasch Analysis – General Partial Credit Model | No global model fit (not unidimensional)  Copying a square and copying a star were misfitting  After removing misfitting items, BOT-2 SF was unidimensional (19.92 ≤ χ2≤ 60.71; 0.06 ≤ P ≤ .97  Disordered thresholds for all items except item 5  Good sensitivity and reliability across the continuum of motor competence for 6‐ to 8‐year‐old children, 9-11 year olds showed ceiling effects | **48%** |
| Barnett et al. (2014) | TGMD-2 | 37 children (65% girls) aged 4–8 years (M = 6.2, SD = 0.8) | Inter-rater reliability | Intraclass Correlation | Stationary dribble:  Contacts ball with one hand at about belt level k1=0.83 k2=0.71  Pushes ball with fingertips (not a slap) k1= 0.62 k2=0.63  Ball contacts surface in front of or to the outside of preferred foot k1=0.94, k2=0.87  Maintains control of ball for four consecutive bounces without having to move the feet to retrieve it – k1=0.75 k2= 0.87  Striking a stationary ball:  Dominant hand grips bat above nondominant hand k1= 0.92 k2=0.91  Nonpreferred side of body faces the imaginary tosser with feet parallel – k1=0.38 k2=1.00  Hip and shoulder rotation during swing k1=0.27 k2= 0.32  Pronounced/clear transfer of body weight to front foot k1=0.68 k2= 0.61  Bat contacts ball k1= 0.88 k2=0.69  Overhand throw:  Windup is initiated with downward movement of hand/arm k1= 0.34 k2= 0.39  Rotates hip and shoulders to a point where the nonthrowing side faces the wall k1=0.50 k2= 0.53  Weight is transferred by stepping with the foot opposite the throwing hand k1=0.42 k2= 0.62  Follow-through beyond ball release diagonally across the body and down towards the non-preferred side k1=0.65 k2= 0.65  Underhand roll  Preferred hand swings down and back, reaching behind the trunk while chest faces cones k1=0.84 k2=0.87  Strides forward with foot opposite the preferred hand towards the cones k1= 0.62 k2= 0.72  Bends knees to lower body k1=0.72 k2=0.49  Releases ball close to the floor so ball does not bounce more than 4 inches high k1=0.59 k2= 0.47  Kick  An elongated stride or leap immediately prior to ball contact k1= 0.84 k2=0.36  Nonkicking foot placed even with or slightly in back of the ball k1=0.69 k2= 0.65  Kicks ball with instep of preferred foot (shoe-laces) or toe k1=1.00 k2=1.00  Catch  Preparation phase where hands are in front of the body and elbows are flexed k1=0.37 k2= 0.54  Arms extend while reaching for the ball as it arrives k1=0.42 k2= 0.53  Ball is caught by hands only k1= 0.89 k2=0.81 | **54%** |
|  |  |  |  | Intraclass correlation | Object control subset= excellent (0.93)  Dribble = 0.94 (0.89-0.97)  Strike= 0.85 (0.73-0.92)  Throw= 0.84 (0.70-0.91)  Roll= 0.82 (0.67-0.90)  Kick= 0.80 (0.64-0.89)  Catch= 0.71 (0.54-0.84) |  |
| Borremans et al (2009) | MABC- 2 | 30 young adults aged 15–21 years (21 males; 9 females; mean age =  17.2; SD = 1.2) with Asperger syndrome  Control group =  30 young adults (mean age= 16.9; SD = 0.8yrs) | \| Internal consistency \| \| --- \| | Cronbach’s Alpha | Manual dexterity= .44 (in both groups)  If bimanual task deleted= .65 for control group; .71 for AS group  Ball skills = .73 (control) .84 (AS group)  Balance = .35 (control group) .57 (AS)  Zigzag hopping items deleted =.73. | **63%** |
|  |  |  | Hypothesis testing validity | MANOVA | AS performed statistically lower compared to control on overall motor competence (Wilk’s Lambda =  .49, F(11, 48) = 4.48 (p < .001), manual dexterity,  **λ** = .72, F(4, 55) = 5.29 (p < .001), ball skill  items **λ** = .63, F(3, 56) = 11.06 (p < .001); and  for balance items **λ** = .65, F(4, 55) = 7.34 (p <  .001). |  |
| Brian et al. (2018) | TGMD-2 and TGMD-3 | 66 children and adolescents (boys = 41; girls = 25) aged  9–18 years (mean age = 12.93, SD = 2.40 years)  White = 51; Black = 9; Asian = 2; Hispanic = 4  Mean body mass  index of 21.78 (SD = 5.85; boys = 22.09, SD = 6.36; girls = 21.32, SD = 5.07). | Inter-rater reliability | Intra-class correlation | TGMD-3:  Gross motor scale= ICC = .91; CI [.85, .94]),  Locomotor= ICC = .92; CI [.87, .95  Ball skills scales = (ICC = .92; CI [.87, .95]) | **44%** |
|  |  |  | Internal consistency | McDonald’s omega and gauge reliability | TGMD-3  Gross motor (ω = .95; , CI, [.93, .96])  Ball skills subscale (ω = .91; CI [.87, .94])    Locomotor subscales (ω = .89; CI [.84, .93]) |  |
|  |  |  | Structural Validity | Confirmatory factor analysis  Pearson product moment correlations | Correlations ranged from r = .98–.99 (all significant)  Factor loadings ranged from .57 to .92.  Ball skill and locomotor subscales= (.89) |  |
|  |  |  | Criterion Validity (Concurrent) | Pearson product moment correlations | Object control TGMD-2 & ball skills TGMD-3= .98  Object control TGMD-2 & Locomotor TMGD-2 = .84  Object control TGMD-2 & Locomotor TMGD-3 = .87  Object control TGMD-2 & Gross Motor TGMD-2 = .96  Object control TGMD-2 & Gross Motor TGMD-3 = .96  Ball skills TGMD-3 & Locomotor TGMD-2 = .86  Ball skills TGMD-3 & Locomotor TGMD-3 = .9  Ball skills TGMD-3 & Gross Motor TGMD-2 = .96  Ball skills TGMD-3 & Gross Motor TGMD-3 = .98  Locomotor TGMD-2 & Locomotor TGMD-3 = .98  Locomotor TGMD-2 & Gross Motor TGMD-2 = .96  Locomotor TGMD-2 & Gross Motor TGMD-3 = .94  Locomotor TGMD-3 & Gross Motor TGMD-2 = .96  Locomotor TGMD-3 & Gross Motor TGMD-3 = .97  Gross Motor TGMD-2 & Gross Motor TGMD-3 = .99 |  |
| Brown (2019) | BOT-2-BF | 123 children aged 8-12 (67 males & 56 females; mean age=10 years, 2 months; SD=1 year, 4  months) | Structural Validity | Rasch Measurement Model Analysis | 14 item BOT-BF:  Bilateral Coordination item 3 (BC3), Bilateral Coordination item 6 (BC6), Balance item 2 (B2), Fine Motor Integration item 7 (FMI7),  Strength item 2 (S2), Upper-limb Coordination item 1 (UC1), Fine Motor Integration item 2 (FMI2), Manual Dexterity item 2 (MD2),  and Fine Motor Precision item 3 (FMP3) were misfitting items  BOT-2 BF was unidimensional  DIF was found for balance item 7 when examining gender differences  Person-separation reliability was 0.63, and  item-separation reliability was 1.00  The Person Raw Score reliability for the BOT-2-BF was 0.69  Revised 5 item BOT-2 BF (nine misfitting items removed)  No misfitting items  No items had DIF based on gender  Unidimensionality requirements were met  Person-separation reliability = 0.60, and item separation  reliability =1.00  Person Raw Score reliability = 0.62. | **60%** |
| Brown (2019b) | BOT-2 | 117 children aged between 8 and 12 years of age - mean age of 10 years 2 months  65 males (56.6%) and  52 females (44.4%) | Structural Validity | Rasch Analysis | Item Fit – logit scores for the eight BOT-2 subscale items ranged from −9.36 to 7.46. Upper limb coordination had one misfitting item. Balance had 4 misfitting items. FMCC and BCC both had a large number of items that didn’t meet Rasch requirements  Unidimensionality - The percentage of unexplained variance in the eight BOT-2 subscales ranged from 0.00% to 4.70%. Subscales are unidimensional. The percentage unexplained for composite scales ranged from .9% - 2%, and are unidimensional  DIF- balance and upper limb coordination had one item which exhibited DIF by gender. MCC and BCC had 2 items with gender DIF.  Reliability - Item reliability of the eight BOT-2 subscales ranged from 0.95 to 1.00 while person  reliability coefficients ranged from 0.08 to 0.80. For the four composite scales, item reliability coefficients ranged from 0.98 to 1.00 and person reliability coefficients  ranged from 0.50 to 0.85. | **93%** |
| Cairney et al. (2009) | BOT-SF   MABC | BOT-SF = 2058 children  24 of 128 children aged 10 (n = 10), 11 (n = 10) or 12 (n = 4) scoring below the sixth percentile = MABC | Concurrent validity | PPV; 5^th^ and 15^th^ percentile cut-points on the M-ABC | 21 of 24 children identified as probable  DCD on the BOT-SF were below the 15th percentile on  M-ABC - PPV = 0.88 (95% CI = 0.69 to 0.96).  15 children were below the 5th percentile – PPV= 0.63 (95% CI = 0.43 to 0.79) | **30%** |
| Cairney et al (2018) | PLAYfun | 215 children in total: 112 (52%) males and 103 (48%) females.  Age was not recorded for one child. For the remaining 214, the average age was 10.3 years (SD=1.7), with a minimum of 6.5 and a maximum of 14.1. | Reliability / Measurement Error  (Inter-rater reliability) | Intra-class correlation (ICC) | For the total score among 7 assessors in the pilot sample of 10 children:  ICC = 0.87 | **76%** |
|  |  |  | Structural Validity  Hypothesis testing validity | Confirmatory factor analysis | The fit of the initial model was fair (RMSEA = 0.065, 90% CI = 0.052 to 0.077; CFI =0.93; TLI = 0.91).  Modification - a path to allow error terms for tasks 15 and  16. RMSEA = 0.055, 90% CI = .03 - .075, CFI = .95, TLI = .94. |  |
| Cano-Cappellacci,Leyton and Carreno (2015)   \|  \| \| --- \| | TGMD-2 Chilean Version  TGMD-2 | 92 children aged 5-10 (56 boys; 36 girls; mean age=7.5; SD=1.6) | Content Validity | Content validity index (CVI) | Language clarity - CVI = 0.88 for TGMD-2-CH  - CVI = 0.83 for the modified  TGMD-2  TGMD-2-CH CVI = 0.90  Modified test CVI = 0.84 | **41%** |
|  |  |  | Inter-Rater Reliability | T-Test | Total score - *p*=.006  Locomotor – *p*=.14  Object Control – *p*=.01 |  |
|  |  |  |  | CVI (95% CI) | Total score - 0.86 (0.72,0.93)  Locomotor - 0.87 (0.73,0.93)  Object Control - 0.88 (0.77,0.94) |  |
|  |  |  | Intra-Rater Reliability | T-Test | Total score - *p*=.91  Locomotor – *p*=.92  Object Control – *p*=.86 |  |
|  |  |  |  | CVI (95% CI) | Total score - 0.91 (0.83, 0.95)  Locomotor - 0.92 (0.83, 0.95)  Object Control - 0.86 (0.76, 0.93) |  |
|  |  |  | Test-Retest Reliability | T-Test | Total score - *p*=.88  Locomotor – *p*=.86  Object Control – *p*=.80 |  |
|  |  |  |  | CVI (95% CI) | Total score - 0.88 (0.75, 0.94)  Locomotor - 0.86 (0.71, 0.93)  Object Control - 0.80 (0.59, 0.90) |  |
| Capio, Eguia and Simons (2016) | TGMD-2 | 81 children with intellectual disability aged 5-14 (65 boys; 16 girls; mean age = 9.29; SD= 2.71 years) | Intra-rater Reliability | Intra-class correlation | Locomotor= 0.995; (0.978-0.999)  Object control= .998 (0.991-0.999)  Total FMS= 0.997 (0.989-0.999) | **56%** |
|  |  |  | Inter-rater reliability | Intra-class correlation | Locomotor= 0.996 (0.984- 0.999)  Object control= 0.998 (0.992-1.000)  Total FMS= 0.998 (0.991-0.999) |  |
|  |  |  | Internal consistency | Cronbach’s alpha | Locomotor components = 0.830; range of 0.757–0.814 when each item is deleted.  Object control components= 0.792; range of 0.713–0.757 when each item is deleted.  Item-total correlation coefficients = locomotor components = 0.712 to 0.913,  Object control components = 0.673 to 0.816. |  |
|  |  |  | Content Validity   \|  \| \| --- \| | Pearson product–moment correlation coefficient | Age and locomotor = (r = 0.222, P = 0.047) and object control skills (r = 0.356, P = 0.001). |  |
|  |  |  |  | Multivariate analysis of covariance (ANCOVA) | Multivariate ANCOVA = (F(2, 78) = 5.865, P = 0.004, η2 = 0.131). |  |
|  |  |  | Structural validity | Confirmatory factor analysis (CFA)  Hypothesis testing validity | Locomotor and object control of TGMD-2 with fit indices: χ2 = 33.525, DF = 34, P = 0.491, χ2/DF = 0.986  GFI = 0.931.  RMSEA = 0.000 with 90% CI of 0.000–0.080 |  |
| Capio, Sit and Abernethy (2011) | TGMD-2 | 30 children with CP (17 girls, 13 boys) aged 6-14 (mean age= 9.83 years, SD = 2.5 years). | Inter-rater reliability | Cohen’s Kappa coefficient | Kappa ranged from 0.875 – 0.907 | **47%** |
| Chow et al. (2002) | \| Age Band IV of the MABC test \| \| --- \| | 31 teenagers (mean age = 13.92, SD= 1.26) | Test- retest reliability | Intraclass correlation coefficient (ICC) | One-hand catch – preferred hand = 0.75  One-hand catch – non-preferred hand = 0.84  Throwing at wall target = 0.76  Two-board balance = 0.73  Cross board balance = 0.91  Jumping and clapping = 0.84  Zig-zag hopping – preferred leg = 0.91    Zig-zag hopping – non-preferred leg = 0.89  Walking backwards =0.06 | **48%** |
|  |  |  | Inter-rater reliability | Intraclass correlation coefficient (ICC) | One-hand catch – preferred hand =0.98  One-hand catch – non-preferred hand =0.97  Throwing at wall target = 0.92  Two-board balance = 1.00  Cross board balance =0.98  Jumping and clapping = 0.52  Zig-zag hopping – preferred leg =0.96  Zig-zag hopping – non-preferred = 0.96  Walking backwards = 0.95 |  |
| Crawford, Willson and Dewey (2001) | BOT & M-ABC | 101 children with DCD (61 boys; 40 girls; mean age= 11.62 yrs; SD = 1.97)  (low SES=25.8%; middle SES= 47.3%; high SES= 26.9%) and  101 matched children in the non DCD group (81 boys; 20 girls; mean age= 11.50; SD= 2.00)  (low SES= 22.1%; middle SES= 51.6%; high SES= 26.3%) | Criterion Validity (Concurrent) | Observed agreement between tests (Po) and  agreement corrected for by chance (kappa) | BOT (gross motor) = PO= 0.846, Kappa= 0.673  BOT (fine motor) and BOT (battery composite) = PO= 0.791, Kappa= 0.476  BOT (fine motor) and BOT (gross motor) = PO= 0.667, Kappa= 0.264  M-ABC and BOT battery composite = PO= 0.722, Kappa= 0.416  M-ABC and BOT Gross Motor= PO= 0.722, Kappa= .430  M-ABC and BOT Fine Motor= PO= 0.569, Kappa= 0.073 | **50%** |
| Croce, Horvat and McCarthy (2001) | MABC,  Bruininks-Oseretsky test | 106 children aged 5-12 (39 girls; 67 boys) | Criterion Validity (Concurrent) | Pearson correlation coefficients | MABC test- Bruininks-Oseretsky Long From  All groups= .76  5-6yr olds= .77  7-8yr olds= .76  9-10yr olds= .70  11-12 yr olds= .90  MABC - Bruininks-Oseretsky Short Form  All groups= .71,  5-6yr olds= .79  7-8yr olds= .76  9-10yr olds= .60  11-12 yr olds= .90 | **40%** |
|  |  |  | Test-retest reliability of the MABC | Intraclass correlation coefficient (ICC) | All groups= .95  5-6 yr olds= .98  7-8yr olds= .95  9-10yr= .92  11-12yr= .97 |  |
| Darsaklis et al. (2013) | M-ABC-2 and BOT | Not specified | Inter-rater reliability | Cohen’s kappa | MABC (overall) = κ = 0.93  BOT:  Running speed and agility= k= 1.00  Balance= k=1.00  Bilateral coordination= k=1.00  Strength= k=1.00  Upper-limb coordination = k = 1.00 | **13%** |
| \| Dos Santos et al. (2017) \| \| --- \| | MABC-2 | 350 Children (188 girls and 162 boys) 350 children (162 boys and 188 girls) aged  between 8 and 10.  (Associação  Brasileira de Empresas de Pesquisa [ABEP], 2008), 1.4% of  the students belonged to social class A2, 8% to social class B1,  27.1% to social class B2, 35.7 % to social class C1, 17.4% to  social class C2, 8.6% to social class C and 1.4% to social class  D. | Structural validity | Confirmatory factor analysis | Correlations between items and MABC-2  Catching with two hands = 0.31  Throwing beanbag onto mat = 0.33  One-board balance for right foot = 0.73  One-board balance for left foot =0.72  Walking heel to toe forwards = 0.34  Hopping on mats 2 for right foot = 0.37  Hopping on mats 2 for left foot = 0.38  1 factor model:  cfd/² = 3.99, GFI = .91, AGFI = .86, CFI= .72, RMSEA =.09  Original 3 factor model:  cfd/² = 2.82, GFI = .96, AGFI = 94, CFI= .97, RMSEA =.06  Schulz et al (2011) model:  cfd/² = 1.97, GFI = .98, AGFI = .97, CFI= 1, RMSEA =.01 | **47%** |
| Ellinoudis and Thomas (2008) | MABC | In total 220 participants - 110 boys and 110 girls  (Mean age in months = 126.5, SD = 3.49)  Participants were divided  into two age groups:  First = boys (n=55) and girls (n=55) aged 9 to 10 years (n=110, Mean age in months = 114.74, SD = 3.88)  Second = boys (n=55) and girls (n=55) aged 11 to 12 years (n=110, Mean age in months = 138.3, SD = 3.11) | Structural validity | Cronbach’s alpha | Age band 3: ranged from .30 to .80.  Age band 4: ranged from .41 to .77 | **54%** |
|  |  |  |  | Pearson correlation coefficients | Correlation between item score and MABC score  Age band 3:  Two-hand catch -.52  Throw bean bag into box -.43  One-board balance-preferred leg -.35  One-board balance-other leg -.39  Hopping in squares-preferred leg -.23  Hopping in squares-other leg -.26  Ball balance .52  Age band 4:  One-hand catch-preferred hand -.50  One-hand catch-other hand -.37  Throw at wall target -.46  Two-board balance -.46  Jump and clap -.59  Walking backwards -.30 |  |
|  |  |  |  | Cohens effect size | Correlation between item score and MABC score  Age band 3:  Two-hand catch -1.2  Throw bean bag into box -1  One-board balance-preferred leg -.75  One-board balance-other leg -.85  Hopping in squares-preferred leg - .5  Hopping in squares-other leg - .55  Ball balance - 1.2  Age band 4:  One-hand catch-preferred hand -1.2  One-hand catch-other hand -.8  Throw at wall target -1  Two-board balance -1  Jump and clap -1.5  Walking backwards- .6 |  |
|  |  |  |  | Principal components factor analysis | Age band 3:  Eigen values greater than 1= 5 factors; explaining 77.38% of the variance.  Factor 1= 23.2% of variance, included “hopping in squares-preferred leg” and “hopping in squares-non preferred leg”. Labelled as "Dynamic Balance".  Factor 2= 17.3% of variance, included “two-hand catch” and “throw bean bag into box”. Labelled "Ball Skills".  Factor 3= 16.0% of variance, included “shifting pegs by rows-preferred hand” and “shifting pegs by rows- non preferred hand”. Labelled "Manual Dexterity 1".  Factor 4 = 10.5% of variance, included “One-board balance- preferred leg” and “one-board balance- non-preferred leg”. Labelled "Static Balance".  Factor 5= 10.19% of variance, included “threading nuts on bolt” and “ball balance”. Labelled "Manual Dexterity 2".  Age band 4:  Eigen values greater than 1= 4 factors; explaining 72.1% of variance.  Factor 1= 27.3% of variance, included “one-hand catch-preferred hand”, “one-hand catch-non preferred hand” and “throw at wall target”. Labelled “ball skills”.  Factor 2= 19.7% of variance, included “turning pegs-preferred hand” and “turning pegs-non preferred hand”. Labelled "Motor Speed on Hand Dexterity".  Factor 3= 12.8% of variance, included “cutting-out elephant” and “flower trail”. Labelled "Motor Accuracy on Hand Dexterity".  Factor 4= 12.2% of variance, included “two-board balance” and “walking backwards”. Labelled "Balance". |  |
| Estevan et al. (2017) | TGMD-3 | 178 typically developed children with an age range between 3 and 11 years (Mean age 6.94 years (SD = 1.89))  Girls = 47.5%  Boys = 52.5%   \|  \| \| --- \| | Internal consistency | ICC | ICC= 0.89 (95% CI, 0.87-0.92) | **57%** |
|  |  |  | Inter-rater reliability | ICC | ICC= 0.90 (95% CI, 0.66-0.98) |  |
|  |  |  | Intra-rater reliability | ICC | ICC= 0.98 (95% CI, 0.85-1.00) |  |
|  |  |  | Structural validity | Maximum likelihood model comparing fit with two-factor model | χ2 (64) = 139.200, p < 0.01, RMSEA = 0.073, SRMR = 0.050, NNFI = 0.964, CFI = 0.970, |  |
| \| Evaggelinou, Tsigilis & Papa (2002) \| \| --- \| | TGMD | \| 644 children (310 girls; 334 boys) Age ranged from 3 to10 years  Participants were divided into two subsamples:  The calibration sample: (n = 324) -  150 males (M age = 7.47 years, SD = 1.59) and 174 females (M age = 7.72 years, SD = 1.69)  The validation sample: (n = 320) - 160 males (M age = 7.68 years, SD = 1.60) and 160  females (M age = 7.68 years, SD = 1.51) \| \| --- \| | Structural validity  Hypothesis testing validity | Confirmatory factor analysis | Locomotor: mean factor loading = .50  Gallop: .51  Hop: .63  Jump: .59  Leap: .56  Run: .27  Skip: .47  Slide: .48  Object control: mean factor loading = .57  Bounce: .80  Catch: .61  Kick: .45  Strike: .41  Throw: .61 | **61%** |
| Ferreira et al (2020) | BOT-2 | 931 (477 girls and 454 boys)  603 children enrolled in public schools and 328 children enrolled in private schools  All aged between 6-10 years old | Cross-Cultural Validity | Percentile curves | Brazilian children showed better results in bilateral coordination, balance, upper-limb coordination, and running speed and agility subtests (difference range 0.03 - 6.90 points).  Upper limb coordination and balance subtests curves were similar. | **46%** |
| Field et al (2020) | TGMD-2 and TGMD-3 | Final sample:  n = 270 (54% female; mean age in grade 3  = 8 years 6 months)  11 children were reported as having a disability or chronic health condition (by their parents) | Reliability / Measurement Error  (Inter-Rater Reliability) | Percent Agreement | Inter-rater reliability between the primary investigator and a second trained research assistant:  TGMD-2 (.88)  TGMD-3 (.87) | **30%** |
|  |  |  | Reliability / Measurement Error  (Intra-Rater Reliability) | Percent Agreement | Intra-rater reliability:  TGMD-2 (.98)  TGMD-3 (.95) |  |
|  |  |  | Criterion Validity (Concurrent Validity) | Paired samples t-tests | Significant difference (p < .05) between the tests (TGMD-2 and TGMD-3) in grade 3  Significant difference (p < .05) between tests in grade 4  Significant difference (p < .05) between tests in grade 5 |  |
| Fransen et al., (2014) | BOT-2 Short Form; KTK   \|  \| \| --- \| | 2485 children  (1300 boys and 1185 girls) aged between 6 and 12 years | Concurrent Validity  Hypothesis testing validity | Pearson correlations | Total BOT-2 Short Form score & KTK motor quotient (r = 0.61, p < 0.001)    BOT- 2 Short Form gross motor composite score & KTK motor quotient (r = 0.44, p < 0.001)    BOT-2 Short Form fine motor composite score and KTK Motor Quotient (r = 0.25, p < 0.001) | **47%** |
| Furtado and Gallagher (2012) | FG-COMPASS   \|  \| \| --- \| | 131 children from 6-11 years    Volunteers from private schools in Western Pennsylvania | Inter-rater reliability | Overall agreement (Ao) | Locomotor:  Hopping: Ao= 87%  Horizontal jumping Ao= 74%  Leaping Ao=66%  Skipping Ao= 82%  Side sliding Ao= 66%  Manipulative:  Batting Ao=82%  Catching Ao: 77%  Kicking Ao= 61%  Overhand throwing Ao=76%  Side-arm striking Ao=84%  Stationary dribbling Ao= 76% | **43%** |
|  |  |  |  | Weighted kappa (Kw) | Locomotor:  Hopping: KW= .85  Horizontal jumping: KW= .70  Leaping: KW= .61  Skipping: KW= .77  Side sliding: KW= .61  Manipulative:  Batting: KW= .79  Catching: KW= .72  Kicking: KW= .51  Overhand throwing: KW= .74  Side-arm striking: KW= .79  Stationary dribbling: KW= .72 |  |
|  |  |  |  | Specific agreement (Ps) | Locomotor:  Hopping: P (I) = .93, P(E) = .79, P(A) = .88  Horizontal jumping: P(I) = .65, P(E) = .63, P(A)= .97  Leaping: P(I) = .70, P(E)= .43, P(A)=.80  Skipping: P(I)= .93, P(E)= .77, P(A)= .74  Side sliding: P(I)= .77, P(E) = .40, P(A)= .73  Manipulative:  Batting: P(I)= .82, P(E)= .75, P(A)=.90  Catching: P(I)= .71, P(E)= .72, P(A)= .88  Kicking: P(I)= 28, P(E) = .56, P(A)= .91  Overhand throwing: P(I)= .74, P(E)= .50, P(A)= .97  Side-arm striking: P(I)= .86, P(E)= .83, P(A)=.83  Stationary dribbling: P(I)= .78, P(E)= .65, P(A)= .84 |  |
| Garn and Webster (2017) | TGMD-2 | 1,120 children between the ages of 3 and 10 years (M age = 7.04, SD = 2.23 years)  49.7% =male  50.3% =female  Racial/ethnic backgrounds:  60.89% White/ Caucasian, 17.59%  Black/ African-American,  14.29% Hispanic, 3.30% Asian,  2.77% Mixed racial/Other, and 1.16% Native  American.  Approximately 7% of the participants had a disability | Structural validity | CFA | 1 factor model:  χ2 = 416.03 (54), p=.001, CFI=.95, RMSEA=.06  2 factor model:  χ2 = 250.24 (53), p=.001, CFI=.97, RMSEA=.05, r=.89 | **79%** |
|  |  |  | Hypothesis Testing Validity | Exploratory SEM | 2 Factor:  χ2 = 164.63 (43), p=.001, CFI=.98, RMSEA=.04, r=.81 |  |
| Hoeber et al. (2018) | Athletic Skills Tract (AST) and KTK | 717 (344 girls and 373 boys) children in study 1  Mean age (SD) = 9 (2) years  213 (104 girls and 109 boys) other children in study 2  Mean age (SD) = 9 (2) years | Test- retest reliability for AST | Intraclass correlation coefficient | AST-1: 0.881 (95% CI: 0.780–0.934)  AST-2: 0.802 (95% CI: 0.717–0.858)  AST-3: 0.800 (95% CI: 0.669– 0.871) | **55%** |
|  |  |  |  | Limits of agreement (LoA) | AST-1: (mean = 0.79, [LoA] −3.02 and 4.60)  AST-2: (mean = 1.47, [LoA] −6.12 and 9.06)  AST-3: (mean = 1.68, [LoA] −5.14 and 8.50) |  |
|  |  |  | Internal consistency of AST | Cronbach’s α | AST-1: α = 0.764  AST-2: α = 0.700  AST-3: α = 0.763 |  |
|  |  |  | Criterion Validity (Concurrent) with KTK | Pearson’s correlation coefficients quotients of the KTK) | AST-1: r = −0.747, p = 0.01  AST-2: r = −0.646, p = 0.01  AST-3: r = −0.602, p = 0.01 |  |
| Hoeboer et al. (2016) | Athletic Skills Track 1 and 2 (AST-1&2)  KTK | 463 children (211 girls, 252 boys)  aged between 6 and 12 years  Mean age = 9 ± 2 years | Criterion Validity (Concurrent) | Pearson correlation | AST-1 and KTK  (r = –0.474, P < 0.01)  AST-2 and KTK  (r = –0.502, P < 0.01)  Gender split AST-1 and KTK:  girls: r = –0.501, P < 0.01; boys: r = –0.533, P < 0.01  Gender split AST-2 and KTK  girls: r = –0.448, P < 0.01; boys: r = –0.566, P < 0.01 | **45%** |
|  |  |  | Test-retest reliability | Intraclass correlation | Between the first and second AST-1 trial = 0.875 (95% CI [0.852–0.895])  Between the first and second AST-2 trial = 0.891 (95% CI [0.870–0.908]) |  |
|  |  |  |  | Paired sample t test | AST-1 trials 1 and 2  t = 6.026, P < 0.05  AST-2 trials 1 and 2  t = 8.226, P < 0.05 |  |
| Holm, Tveter, Aulie & Stuge (2013) | MABC-2 | 45 healthy children s (7–9 years of age)  Females = 23  Males = 22  Mean age (SD) = 8.7 (0.7) yrs  43 (95.5%) children had no movement problems and 2 (4.5%) children were classified as having impaired motor problems  30 children were included in the inter-tester part  of the study, 29 children in the intra-tester part, 14 took part in both studies | Intra-tester reliability | Intraclass correlation (with 95% Cl and SEM) | Aiming and catching:  Catching with two hands, no. of catches = 0.48 [0.15,0.72], SEM = 1.5  Throwing bean bag on to mat = 0.59 [0.29,0.79], SEM = 1.0  Balance:  One-board balance, right leg = 0.56 [0.26,0.77], SEM = 4.0  One-board balance, left leg = 0.70 [0.45,0.85], SEM = 5.3  Walking heel-to-toe forwards = 0.75 [0.53,0.87], SEM = 0.9  Hopping on mats, right leg = NA  Hopping on mats, left leg = 0.24 [-0.15,0.56], SEM = 0.6  Domains (component score):  Aiming and catching = 0.49 [0.17,0.72], SEM = 2.4  Balance = 0.49 [0.15,0.72], SEM = 2.7  Total score:  Total test score = 0.68 [0.28,0.85], SEM = 4.9  Total standard score = 0.64 [0.23,0.84], SEM = 1.4 | **43%** |
|  |  |  | Inter-rater reliability | Intraclass correlation  (with 95% Cl and SEM) | Aiming and catching:  Catching with two hands = 0.66 [0.40,0.82], SEM = 1.3  Throwing bean bag on to mat = 0.62 [0.33,0.80], SEM = 1.1  Balance:  One-board balance, right leg = 0.39 [0.05,0.65], SEM = 5.8  One-board balance, left leg = 0.50 [0.19,0.73], SEM = 7.3  Walking heel-to-toe forwards = 0.42 [0.06,0.67], SEM = 1.6  Hopping on mats, right leg = NA  Hopping on mats, left leg = NA  Domains (component score):  Manual dexterity = 0.63 [0.35,0.80], 3.2  Aiming and catching = 0.77 [0.56,0.89], SEM = 2.0  Balance = 0.29 [-0.07,0.58], SEM = 4.5  Total score:  Total test score = 0.62 [0.35,0.80], SEM = 6.8  Total standard score = 0.63 [0.36,0.80], SEM = 1.6 |  |
| Houwen et al (2010) | TGMD-2 and MABC | 75 children with VI  (aged between 6 and 12 years, 29 girls; 46 boys)  Of these 75 children, 8 children attended  special-needs schools and 67 children attended mainstream primary schools.  71 children were Caucasian while 4 were of Asian descent. | Internal  consistency | Cronbach’s Alpha | Locomotor= 0.71  Item deleted= 0.61-0.68  Item-total correlations= 0.36-0.54  Inter-item correlations= 0.11 to 0.45  Object control= 0.72  Item deleted= 0.63-0.71  Item-total correlations= 0.30-0.54  Inter-item correlations= 0.14 to 0.51 | 64% |
|  |  |  | Inter-rater reliability TGMD | ICCs (95% CIs) | Locomotor= 0.82 (0.70-0.90)  Object control=0.93 (0.88-0.96)  Total test=0.89 (0.81-0.93) |  |
|  |  |  | Intra-rater reliability  TGMD | ICC | Locomotor= 0.85 (0.69-0.93)  Object control = 0.93 (0.84-0.97)  Total test= 0.95 (0.88-0.98) |  |
|  |  |  | Test-retest reliability  TGMD | ICC | Locomotor= 0.86 (0.70-0.94)  Object control= 0.87 (0.72-0.94)  Total test= 0.92 (0.82-0.91) |  |
|  |  |  | Structural validity | Factor analysis | Fit Indices: Chi-square (Df = 53) = 79.55, *p* = 0.01,  Df ratio = 1.50, RMSEA = 0.07, GFI = 0.85.  Locomotor factor loadings  Run=.50 (p < .05)  Gallop=.44 (p < .05.)  Hop=.49 (p < .05.)  Leap=.61 (p < .05.)  Jump=.51 (p < .05.)  Slide=.76 (p < .05.)  Object control skills factor loadings Strike= .32 (p < .05.)  Dribble= .73 (p < .05.) Catch= .57 (p < .05.) Kick= .62 (p < .05.) Throw= .68 (p < .05.) Roll= .61(p < .05.)  Correlation between LOC and OC= 0.81 |  |
|  |  |  | Criterion Validity (Concurrent) TGMD and MABC | Spearman Rho | TGMD-2 object control subtest and the Movement ABC ball skills subtest  Age band 2= rs = 0.57, *p* = 0.001  Age band 3= rs = 0.45, *p* = 0.040 |  |
| Hua, Gu, Meng & Wu (2013) | MABC-2 and PDMS-2 | 1823 children in total (Females = 908  and Males = 915)  Aged 36–72 months old (mean = 61.284 months, SD = 10.212 months) | Internal consistency  MABC | Cronbach's alpha | Catching beanbag = .428  Throwing beanbag onto mat = .427  One leg balance = .445  Walking heels raised = .517  Jumping on mats = .489 | **67%** |
|  |  |  |  | Pearson correlation coefficients (Item-total correlation) | Catching beanbag = .587  Throwing beanbag onto mat = .603  One leg balance = .525  Walking heels raised = .228  Jumping on mats = .405 |  |
|  |  |  | Inter-rater  reliability | Intraclass correlation  (with 95% Cl) | Catching beanbag (number of correct catches out of 10) = .993 (.992, .994)  Throwing beanbag onto mat (Number of correct catches out of 10) = .979 (.977, .981)  One-leg balance with preferred leg (number of seconds balanced) = .997 (.997, .998)  One-leg balance with non-preferred leg (number of seconds balanced) = .998 (.997, .998)  Walking heels raised (number of correct steps) = .895 (.886, .904)  Jumping on mats (number of correct jumps/hops out of 5) = .993 (.993, .994) |  |
|  |  |  | Test-retest reliability | Intraclass correlation (with 95% Cl) | Catching beanbag (number of correct catches out of 10) = .934 (.912, .950)  Throwing beanbag onto mat (Number of correct catches out of 10) = .905 (.874, .928)  One-leg balance with preferred leg (number of seconds balanced) = .970 (.959, .977)  One-leg balance with non-preferred leg (number of seconds balanced) = .985 (.979, .988)  Walking heels raised (number of correct steps) = .832 (.781, .871)  Jumping on mats (number of correct jumps/hops out of 5) = .936 (.916, .952) |  |
|  |  |  | Content validity | Item-level content validity index | Catching beanbag (number of correct catches out of 10) = 1.0  Throwing beanbag onto mat (Number of correct catches out of 10) = 1.0  One-leg balance (number of seconds balanced) = 1.0  Walking heels raised (number of correct steps) = .96  Jumping on mats (number of correct jumps/hops out of 5) = .96  The average = .985 |  |
|  |  |  | Structural validity | Confirmatory Factor Analysis | Original 8 item model (Henderson, 2007)  x2 = 80.149, df = 17, x2/df = 4.715, p < 0.001, GFI = 0.976, AGFI = 0.950, IFI = 0.850, CFA = 0.846, RMSEA = 0.067  7 item model (heels raised removed)  x2 = 35.828, df = 11, x2/df = 3.257, p < 0.001, GFI = 0.988, AGFI = 0.969, IFI = 0.935, CFA = 0.933, RMSEA = 0.043  6 item model (drawing trail removed)  x2 = 11.749, df = 6, x2/df = 1.958, p = 0.068, GFI = 0.995, AGFI = 0.984, IFI = 0.984, CFA = 0.984, RMSEA = 0.034). |  |
|  |  |  | Criterion Validity (Concurrent) | Spearman's correlation | MABC-2 gross motor (aiming and catching):  PMDS-2 gross motor = 0.743  PMDS-2 total = 0.628  MABC-2 balance:  PMDS-2 gross motor = 0.066  PMDS-2 total = 0.165  MABC-2 total:  PMDS-2 gross motor = 0.457  PMDS-2 total = 0.631 |  |
| Iatridou & Dionyssiotis (2013) | BOT (balance subtest) | 20 children with Cerebral Palsy from Special Education Schools in Greece  6-14 years  Females = 8  Males = 12  Diagnosis of hemiplegia, or diplegia with the capacity of self-support walking  8 children has diplegia | Test-retest reliability | Intraclass correlation | Between the 1st-2^nd^ = 0.978 (p<.001)  Between the 1st-3rd = 0.993 (p<.001)  Between the 2nd-3rd = 0.989 (p<.001)  Between the three measurements (1st, 2nd and 3rd) 3=0.987 (p<.001) | **57%** |
| Issartel, McGrane, Fletcher, O’Brien, Powell, Belton et al. (2017) | TGMD-2 | In total 844 participants (males = 456, females = 388)  Aged 12.03  years ± 0.49 (median = 12.89) | Test-retest reliability | Pearson product moment correlation | Locomotor = 0.78  Object related = 0.76  Gross motor skills = 0.91 | **47%** |
|  |  |  | Structural Validity | Confirmatory Factor Analysis | Full model two correlated factors  χ^2^= 175.26 (53), p<.001, CFI=.59, RMSEA = .05  Full one factor model:  χ^2^= 187.24 (54), p<.001, CFI=.7, RMSEA = .05  Reduced model one factor  χ^2^= 111.29 (35), p<.001, CFI=.68, RMSEA = .05  Reduced model two correlated factors  χ^2^= 87.11 (34), p<.001, CFI=.77, RMSEA = .04 |  |
| Jaikaew & Satiansukpong (2019) | MABC-2, Thai version | 30 children were recruited for the inter-rater reliability study  5 children were recruited for testing MABC-2  All aged 7 years and 0 months to 10 years and 11 months with no “physical limitations” | Reliability / Measurement Error  (Inter-rater reliability) | Intraclass Correlation Coefficient (ICC with 95% CI) | Aiming and Catching:  Catching with Two Hands 0.99 (0.98-0.99)  Throwing a Beanbag onto a Mat 0.94 (0.88-0.97)  Balance:  One-Balance Board (other leg) 1.00 (0.99-1.00)  Walking Heel-to-Toe Forwards 0.99 (0.99-0.99)  Hopping on Mats (best leg) NA  Hopping on Mats (other leg) 0.96 (0.92-0.98) | **29%** |
|  |  |  | Content validity | Item objective congruence  (IOC) index | Manual Dexterity with:  Language clarity (.73)  Language pertinence (.83)  Aiming and Catching with:  Language clarity (.88)  Language pertinence (.95)  Balance with:  Language clarity (.89)  Language pertinence (.94) |  |
| Jirovec, Musalek & Mess (2019) | BOT-2 (CF) and BOT-2 (SF) | 153 children aged 8 to 11 (M = 9.53 ± 0.85 years), (boys n = 84, girls n = 69) | Criterion Validity (Concurrent Validity) short form and complete form | Pearson’s product moment correlations | Manual coordination: 0.24*  Body coordination: 0.42**  Strength and agility: 0.08 | **54%** |
|  |  |  |  | Receiver Operating Characteristic (ROC) analysis of BOT-2 SF | BOT-2 SF:  High sensitivity (84%)  Poor specificity (42.9%)  Accuracy (76.5%)  Poor value of Empirical Area Under Curve Analysis (AUC) = 0.484 CI95% (0.31–0.62)  ROC analyses conducted for boys and girls separately for BOT-2 SF:  High sensitivity (boys = 82.6%, girls = 85.7%)  Low specificity (boys = 53%, girls = 30.7%) |  |
| Kim, Kim, Valentini & Clark (2014) | TGMD-2 | 141 children aged from 3 to 10 years old  Mean age (SD) = 6.8 (1.9) years | Internal consistency | Cronbach’s alpha coefficient | Between the two subtests = .87  Locomotor subset = .82  Object control subset = .73 | **57%** |
|  |  |  | Inter-rater reliability | Pearson’s correlation | Summed score:  Testers A x B = .94**  Testers A x C = .87**  Tetsers B x C = .86*  Score from locomotor subtest:  Testers A x B = .89**  Testers A x C = .81**  Testers B x C = .83**  Score from object control subtest:  Testers A x B = .92**  Testers A x C = .87**  Testers B x C = .86**  Testers A x B x C = .92** |  |
|  |  |  |  | Intraclass correlation | Summed score:  Testers A x B = .97***  Testers A x C = .93***  Testers B x C = .92***  Testers A x B x C = .96***  Score from locomotor subtest:  Testers A x B = .94***  Testers A x C = .90***  Testers B x C = .91***  Testers A x B x C = .94***  Score from object control subtest:  Testers A x B = .85**  Testers A x C = .80*  Testers B x C = .77* |  |
|  |  |  | Test-retest reliability | Pearson’s correlation | Between the raw scores of the locomotor subset = .90, p < .0001  Between the raw scores of the object control subtest = .85, p < .0001 |  |
|  |  |  | Structural validity | Confirmatory factor analysis | 2 factor model:  x2(54) = 86.59, p = .003 CFI =0.94, TLI =0.93, NFI =0.87, GFI = 0.91, and IFI = 0.95 |  |
| Kim, Park & Kang. (2012) | TGMD-2 | 22 children in total with intellectual disabilities (aged between 8.6 to 11.2 yrs, Mean age 9.9 (± 1.3) years)  16 = boys  6 = girls | Inter-rater reliability | ICC | Locomotor skills:  Kicking= 0.85 (0.65, 0.50)  Striking a stationary ball= 0.91 (0.78, 0.75)  Underhand roll= 0.94 (0.83, 0.79)  Overhand throw= 0.93 (.81, 0.78)  Stationary bouncing= 0.89 (0.72, 0.70)  Catch= 0.88 (0.72, 0.53)  Subtotal score= 0.90 (.75, 0.71)  Object control skills:  Hop= 0.93 (0.82, 0.79)  Horizontal jump= 0.92 (0.80, 0.70)  Slide= 0.94 (.84, 0.81)  Run=0.89 (0.73, 0.72)  Gallop= 0.95 (0.86, 0.85)  Leap= 0.91 (0.78, 0.68)  Subtotal score= 0.93 (0.80, 0.78)  Total score= 0.91 (0.78, 0.75) | **54%** |
| Kita, Suzuki, Hirata, Sakihara, Inagaki & Nakai (2016) | MABC-2 | 132 children in total (Females = 48 and Males = 84)  Aged from 7.0 to 10.8 years (mean = 8.8 and standard  deviation (SD) = 1.2)  58, 29, and 45 children were recruited from urban, middle-urban, and rural areas respectively | Internal consistency | Item-total correlation | Catching with Two Hands = 0.536  Throwing Beanbag onto Mat = 0.504  One-Board Balance = 0.573  Walking Heel-to-Toe Forwards = 0.534  Hopping on Mats = 0.480 | **81%** |
|  |  |  |  | Cronbach’s alpha | Catching with Two Hands = 0.557  Throwing Beanbag onto Mat = 0.581  One-Board Balance = 0.531  Walking Heel-to-Toe Forwards = 0.546  Hopping on Mats = 0.537 |  |
|  |  |  | Structural validity | Confirmatory factor analysis | χ^2^ (17) = 12.685, p=.757; v2/df=0.746; GFI=.977; AGFI = .951; CFI = .999; RMSEA = .000 |  |
| Lander, Morgan, Salmon, Logan & Barnett (2017) | CAMSA & Victorian FMS | 34 children in total (all female)  Mean age (± SD) = 12.6 years (± 0.04)  Demographics:  17 = Australian  9 = Asian  8 = European | Test-retest reliability | Intraclass correlation (with 95% Cl) | CAMSA: total score = 0.91 (Cl = 0.83,0.95)  CAMSA: time score = 0.80 (Cl = 0.63,0.89)  CAMSA: skill score = 0.85 (Cl =0.73,0.92)  Victorian FMS = 0.79 (Cl = 0.62,0.89) | **49%** |
|  |  |  |  | Bland–Altman | CAMSA - mean −1.29, [LoA] −5.62 and 3.04  Victorian FMS Assessment - mean −0.38, [LoA] −6.82 and 6.06 |  |
|  |  |  |  | Bivariate correlation | CAMSA - r = 0.02, p = 0.89  Victorian FMS Assessment instrument - r = −0.12, p = 0.49 |  |
|  |  |  | Criterion Validity (Concurrent) | Spearman’s Rho | Between the finishing position of students in the CAMSA using their total CAMSA score and Victorian FMS Assessment in Test 1:  rs = 0.68, p = <0.05  When isolating the skill score of the CAMSA, with the total skill score of the Victorian FMS Assessment:  rs = 0.60, p = <0.05 |  |
| Lane & Brown (2015) | BOT-2 and MABC-2 | 50 typically developing children aged 7–16 years.  The sample was divided into two age bands:  AB2 (7–10 years): 25 children (14 females and 11 males). Mean age of 8 years 11 months  (SD = 1 year, 1 month)  AB3 (11–16 years): 25 children (11 females and 14 males). Mean age of 13 years  4 months (SD = 1 year 8 months).  . | Criterion validity (concurrent) | Spearman’s rho | MABC Age Band 2 – Balance:  BOT bilateral coordination = -.1  BOT Balance = .11  BOT Running speed & agility = .14  BOT strength = .37  BOT body coordination =.13  BOT strength and agility = .32  MABC Age Band 2 – Aiming and catching  BOT bilateral coordination = -.08  BOT Balance = .35  BOT Running speed & agility = .14  BOT strength = -.1  BOT body coordination = .17  BOT strength and agility = .17  MABC Age Band 3– Balance:  BOT bilateral coordination = .15  BOT Balance = .31  BOT Running speed & agility = .45  BOT strength = .51  BOT body coordination =.29  BOT strength and agility = .45  MABC Age Band 3 – Aiming and catching  BOT bilateral coordination = .26  BOT Balance = .01  BOT Running speed & agility = .25  BOT strength = .44  BOT body coordination = .03  BOT strength and agility = .44 | **77%** |
| Laukkanen et al (2020) | KTK | Pooled data from four independent studies in:  Finland (1)  Mean age 6.64 ± 0.36 years, range 1.8 years, n = 278  Finland (2):  Mean age 8.60 ± 0.85 years, range 3.3 years, n = 412  Belgium:  Mean age 8.25 ± 1.09, range 4 years, n = 1896  Portugal:  Mean age 8.31 ± 1.02, range 3.9 years, n = 758 | Internal consistency | Cronbach's alphas | Cronbach's alphas of the KTK test items:  Finland (combined) 0.828  Belgium 0.804  Portugal 0.777 | **51%** |
|  |  |  |  | Item‐total correlations | The corrected item‐total correlations for the norm‐based values of test items: Finland  Walking backwards .571  Hopping for height .710  Jumping sideways .695  Moving sideways .655  The corrected item‐total correlations for the norm‐based values of test items: Belgium  Walking backwards .549  Hopping for height .656  Jumping sideways .687  Moving sideways .588  The corrected item‐total correlations for the norm‐based values of test items: Portugal  Walking backwards .648  Hopping for height .578  Jumping sideways .680  Moving sideways .616 |  |
| Liao, Mao & Hwang (2001) | BOT | 20 TD children  mean = 10.6 years, SD= 2.3 years  6 males, 14 females | Test-retest reliability | % agreement | One-leg standing on floor – 100  On balance beam – 80  on balance beam, eyes closed – 40  Walking forward on walking line – 100  On balance beam - 70  heel-to-toe on walking line - 50  heel-to-toe on balance beam – 50  Stepping over stick on balance beam - 50 | **35%** |
| Logan, Barnett, Goodway & Stodden  (2017) | TGMD-2 and GSGA | 170 children  in total aged between 4 and 11 years old.  (Females = 86  Males = 84)  Participants were Hispanic (n = 94), Caucasian  (n = 70), African American (n = 5) and Native American (n = 1).  Participants were split into the following age groups:  4–5 year olds: (n = 55, boys = 23, girls = 32). Mean age (SD) = 5 (0.54) years  7–8 year olds: (n = 61, boys = 33, girls = 28). Mean age (SD) = 8.1 (0.62) years  10–11 year olds: (n = 54, boys = 28, girls = 26). Mean age (SD) = 10.7 (0.42) years | Criterion Validity (Concurrent) | Spearman's rho  (* and ** indicate significance at the .05 and .01 levels, respectively) | Jump:  TGMD-2:  4-5 years old = .46**  7-8 years old = .26*  10-11 years old = .47**  GSGA:  4-5 years old = .53**  7-8 years old = 0.17  10-11 years old = .41**  Hop:  TGMD-2:  4-5 years old = .65**  7-8 years old = .41**  10-11 years old = 0.25  GSGA:  4-5 years old = .88*  7-8 years old = .48**  10-11 years old = .47**  Throw:  TGMD-2:  4-5 years old = .30*  7-8 years old = .47**  10-11 years old = .62**  GSGA:  4-5 years old = .29*  7-8 years old = .45**  10-11 years old = .71** | **55%** |
|  |  |  |  | Cochran’s Q tests | Assessments differed in classifying:  standing long jump Q(2) = 14.1, P < .01  Hopping Q(2) = 67.2, P < .001  Throwing Q(2) = 100.2, P < .001) |  |
| Logan, Robinson, Rudisill, Wadsworth& Morera  (2014) | TGMD-2 and GSGA | 65 children in total (Females = 33 and Males = 32)  Kindergarten: (n = 20, 10 boys, 10 girls, mean age = 5.7 + 0.38 years)  First grade: (n = 22, 13 boys, 9 girls, mean age = 6.7 + 0.34 years)  Second grade: (n = 23, 9  males, 14 females, mean age = 7.8 + 0.46 years)  Demographics: 72.3% =  African-American,  20% = Hispanic, 7.7% = Caucasian | Criterion Validity (Concurrent) | Spearman correlations | 4-5 yrs  Jump = .5  Hop = .68  Throw = .59  7-8 yrs  Jump = .48  Hop = .51  Throw = .66  10-11 yrs  Jump = .17  Hop = .47  Throw = .7 | **55%** |
| Longmuir et al (2017) | CAMSA | 1165 children  Females = 598  males = 567  8-12 years | Test-retest reliability | ICC | Completion time across short (n = 59; ICC = 0.84; 95%CI: 0.74 to 0.91) and long (n = 16; ICC = 0.82; 95%CI: 0.53 to 0.93) test intervals | **77%** |
|  |  |  | Inter-rater reliability | ICC | Skill score:  All trials = 0.69 (Cl = 0.61, 0.76)  Trial 1 = 0.70 (Cl = 0.61, 0.79)  Trial 2 = 0.66 (Cl = 0.55, 0.77)  Completion time:  All trials = 0.997 (Cl = 0.995, 0.998)  Trial 1 = 0.997 (Cl = 0.994, 0.998)  Trial 2 = 0.993 (Cl = 0.990, 0.995) |  |
|  |  |  | Intra-rater reliability | ICC | Skill Score:  All examiners = 0.52 (Cl = 0.43, 0.60)  Examiner 1 = 0.45 (Cl = 0.20, 0.64)  Examiner 2 = 0.55 (Cl = 0.33, 0.72)  Examiner 3 = 0.43 (Cl = 0.19, 0.63)  Examiner 4 = 0.52 (Cl = 0.28, 0.69)  Examiner 5 = 0.49 (Cl = 0.26, 0.67)  Examiner 6 = 0.57 (Cl = 0.35, 0.73)  Examiner 7 = 0.53 (Cl = 0.30, 0.70)  Completion time  All examiners = 0.996 (Cl = 0.995, 0.997)  Examiner 1 = 0.999 (Cl = 0.999, 1.000)  Examiner 2 = 0.998 (Cl = 0.998, 0.999)  Examiner 3 = 0.991 (Cl = 0.986, 0.994)  Examiner 4 = 0.996 (Cl = 0.994, 0.997) |  |
| Lopes, Saraiva, & Rodrigues (2018) | TGMD-2 | 330 children in total (Females = 164 and Males = 166)  Aged between 5–10 years of age (Mean age with SD = 7.9 ± 1.3) | Test–retest Reliability | Bland-Altman analysis | 95% limits of agreement ranged from 0.80 to 1.13, agreement ratio = 0.96 (0.09).  Locomotor - 95% limits of agreement ranged between 0.85 and 1.17, agreement ratio= 1 (0.08).  Object Control- 95% limits of agreement ranged between 0.63 and 1.16, agreement ratio = 0.80 (0.13). | **62%** |
|  |  |  | Inter-rater reliability | Kappa | Ranged .7 - 1 |  |
|  |  |  | Internal consistency | Cronbach's alpha | Whole test = .69  Locomotor = .46  Object control = .64 |  |
|  |  |  | Structural validity  Hypothesis testing validity | CFA 2 factor model | CFI = .956, NFI = .868, NNFI = .937, SRMR = .048, RMSEA = .036 (90% CI: .010–.054)  All loading coefficients were significant (p < .05), with factor loadings ranging from .31 to .76.  High correlation (r = .77; p < .05) between the two factors |  |
| Lucas et al. (2013) | BOT-2 | 30 participants  Females = 12  Males = 18  Aboriginal and Torres Straight Island Background  Mothers in the sample drank alcohol during pregnancy | Inter-rater reliability | Intraclass correlation | Bilateral co-ordination:  Jumping in place (same sides synchronized) = .34  Tapping feet and fingers (same sides synchronized) = N/A  Balance:  Walking forward on a line = N/A  Standing on one leg on a balance beam (eyes open) = .54  Running speed and agility:  One legged stationary hop = .49  Upper-limb co-ordination:  Dropping and catching a ball (both hands) = 1.00  Dribbling a ball (alternating hands) = .85  BOT-2 score sheet outcomes:  Total point score (Raw) = .92  Standard score (standardized for gender and age) = .89  Percentile rank (%) = .88 | **83%** |
|  |  |  | Test-retest reliability | Intraclass correlation | Bilateral co-ordination:  Jumping in place (same sides synchronized) = -0.066  Tapping feet and fingers (same sides synchronized) = -0.032  Balance:  Walking forward on a line = N/A  Standing on one leg on a balance beam (eyes open) = .17  Running speed and agility:  One legged stationary hop = .25  Upper-limb co-ordination:  Dropping and catching a ball (both hands) = -0.041  Dribbling a ball (alternating hands) = .023  BOT-2 score sheet outcomes:  Total point score (Raw) = .62  Standard score (standardized for gender and age) = .73  Percentile rank (%) = .71 |  |
| Maeng et al. (2017) | TGMD-3 | 10 typically developing children (6 boys and 4 girls)  Age ranged from 3 years, 7 months to 10 years, 9 months old (Mean age = 6.57, SD = 2.51 years)  Demographics:  70% = White 30% = African American | Inter-rater reliability | ICCs (95% CIs) | Locomotor skills:  Run= 0.66 (0.39 to 0.88)  Gallop= 0.66 (0.39 to 0.88)  Hop= 0.92 (0.82 to 0.98)  Skip= 0.90 (0.78 to 0.97)  Horizontal jump= 0.81 (0.61 to 0.94)  Slide= 0.67 (0.41 to 0.88)  Subscale score= 0.92 (0.82 to 0.98)  Ball skills:  Two-handed strike= 0.81 (0.61 to 0.94)  One-handed strike= 0.86 (0.70 to 0.96)  One-handed dribble= 0.92 (0.81 to 0.98)  Two-handed catch= 0.67 (0.41 to 0.88)  Kick= 0.51 (0.22 to 0.80)  Overhand throw= 0.78 (0.57 to 0.93)  Underhand throw= 0.79 (0.59 to 0.93)  Subscale score= 0.93 (0.84 to 0.98)  Total score= 0.96 (0.91 to 0.99) | **61%** |
|  |  |  | Intra-rater reliability | ICC (95% Cis) | Locomotor skills:  Run= 0.91 (0.84 to 0.95)  Gallop= 0.86 (0.76 to 0.92)  Hop= 0.93 (0.88 to 0.96)  Skip= 0.95 (0.91 to 0.97)  Horizontal jump= 0.90 (0.83 to 0.94)  Slide= 0.84 (0.73 to 0.90)  Subscale score= 0.98 (0.96 to 0.99)  Ball skills:  Two-handed strike= 0.86 (0.77 to 0.92)  One-handed strike= 0.92 (0.87 to 0.96)  One-handed dribble= 0.95 (0.92 to 0.97)  Two-handed catch= 0.87 (0.79 to 0.93)  Kick= 0.77 (0.63 to 0.87)  Overhand throw= 0.93 (0.87 to 0.96)  Underhand throw= 0.87 (0.78 to 0.93)  Subscale score= 0.96 (0.94 to 0.98)  Total score= 0.98 (0.96 to 0.99) |  |
| Magistro et al. (2020) | TGMD-3 | 5210 children age range of 3-11; mean age years = 8.38, SD = 1.97; % females = 48.  . | Test-retest reliability | Intraclass Correlations | 6 years old group (n = 50):  Locomotor = (ICC = .993; CI [.987, .996]),  Ball skills scales = (ICC = .992; CI [.986, .995]),  TGMD-3 total= ICC = .991; CI [.983, .995]),  7 years old group (n = 50):  Locomotor = ICC = .983; CI [.971, .990]),  Ball skills scales = (ICC = .989; CI [.981, .984]),  TGMD-3 total= ICC = .979; CI [.964, .988]),  8 years old group (n = 50): Locomotor = ICC = .985; CI [.974, .992]), Ball skills scales = (ICC = .993; CI [.987, .996]), TGMD-3 total= ICC = .981; CI [.967, .989]), 9 years old group (n = 50): Locomotor = ICC = .991; CI [.985, .995]), Ball skills scales = (ICC = .995; CI [.991, .997]), TGMD-3 total= ICC = .989; CI [.980, .993]), 10 years old group (n = 50): Locomotor = ICC = .990; CI [.983, .994]), Ball skills scales = (ICC = .996; CI [.993, .998]), TGMD-3 total= ICC = .993; CI [.987, .996]), 11 years old group (n = 50): Locomotor = ICC = .982; CI [.968, .990]), Ball skills scales = (ICC = .994; CI [.989, .996]), TGMD-3 total= ICC = .984; CI [.972, .991]) | **64%** |
|  |  |  | Inter-rater reliability | Intraclass correlation  coefficients | TGMD-3 total scores = 0.973; 95% CI: Lower Bound = 0.969 and Upper Bound = 0.977). |  |
|  |  |  | Structural validity | Exploratory and confirmatory factor analysis | CFA with ML estimation method  χ2= 916.284, df = 64, p < 0.001, RMSEA = 0.050 (90% Confidence Intervals: 0.048, 0.053), CFI = 0.955.  Factor loadings were all significant at p < 0.001 and ranged between 0.583–0.671.  Locomotor Skills Run: EFAβ= .323 / CFAβ= .671 Gallop: EFAβ= .363 / CFA β= .615 Hop: EFA β= .405 / CFA β= .675 Skip: EFA β= .426 / CFA β= .584 Horizontal jump: EFA β= .426 / CFAβ= .622 Slide: EFA β= .454 / CFA β= .585  Ball skills Forehand strike of self-bounced ball: EFA β= .387 / CFA β= .565 One-hand stationary dribble: EFA β= .433 / CFA β= .656 Two-hand catch: EFAβ= .374 / CFA β= .604 Kick a stationary ball: EFA β= .244 / CFA β= .629 Overhand throw: EFA β= .421 / CFA β= .603 Underhand throw: EFA β= .353 / CFA β= .589 Two-hand strike of a stationary ball: EFA β= .376 / CFA β= .597 |  |
| Mancini, Rudaizky, Howlett, Elizabeth-Price & Chen (2019) | BOT-2 | 86 children with ADHD. 78 males and 6 females aged 6-14 years (M = 9 years, 11 months; SD = 1 year, 9 months). | Criterion Validity  (Concurrent) (Long- and short-form BOT-2) | Pearson's bivariate correlation | Correlation with domain score  Jumping in place-same sides synchronised = .561*  Tapping feet and fingers-same sides synchronised = .587*  Walking forward on a line = .173  Standing on one leg on a balance beam - eyes open = .122  One-legged stationary hop = .676*  Dropping and catching a ball - both hands = .333*  Dribbling a ball - alternating hands = .323*  *p<.001. | **53%** |
| Moreira, Lopes, Miranda-Junior, Valentini, Lage & Albuquerque (2019) | KTK | 565 volunteers from 5 to 10 years of age (age mean = 7.93 ±1.51. 49.9% were boys (n= 282) and 50.1% were girls (n= 283), all whom are enrolled in Brazilian public and private schools (from 1^st^to 5^th^grade of elementary school). | Structural Validity | Confirmatory factorial analysis (CFA) | χ2 = 5.086, *p* = 0.079, CFI = 0.995, TLI = 0.986, RMSEA = 0.052, SRMR = 0.015).  CFA for male group (χ2 = 2.733, *p* = 0.255, CFI = 0.998, TLI = 0.993, RMSEA =0.036, SRMR = 0.016)  CFA for female group (χ2 = 3.255, *p*= 0.196, CFI = 0.997, TLI = 0.990, RMSEA = 0.047, SRMR = 0.016).  CFA for 5 to 7 years old group (χ2 = 0.340, *p*= 0.844, CFI = 1.000, TLI = 1.020, RMSEA = 0.000, SRMR = 0.006)  CFA for 8 to 10 years old group (χ2 = 5.881, *p*= 0.053, CFI = 0.981, TLI = 0.943, RMSEA = 0.076, SRMR = 0.027). | **59%** |
| Nicola, Waugh, Charles & Russell (2018) | MABC-2 | Final sample: n=59, aged 5–11 years  (Females n = 28 and Males n = 31)  The ABs were as follows: 3–6 years (n=19), 7–10 years  (n=31); and 11–16 years (n=9) | Criterion Validity (Concurrent) MABC-2 in person and via telerehabiliation technology  Hypothesis testing validity | Mean absolute difference (SD) | Aiming & Catching = 0.27 (2.07)  Balance = 0.15 (2.59)  Total Test Score = 0.03 (1.63) | **40%** |
|  |  |  |  | Percentage Agreement | % Exact; % within 1 point; % within 2 points; % within 3 points  Aiming & Catching = 26.67; 51.67; 71.67; 90  Balance = 31.67; 51.67; 71.67; 81.67  Total Test Score = 31.67; 66.67; 81.67; 100 |  |
|  |  |  |  | Bland-Altman | Upper & Lower limits  Aiming & Catching = 3.80, −4.33  Balance = 5.23, −4.93  Total Test Score = 3.22, −3.15 |  |
|  |  |  |  | Paired samples T-test  (95% Cl) | Aiming & Catching: TR vs in-person  = 0.27 (SD = 2.09), (Cl = -0.82, 0.27), P = 0.32  Balance: TR vs in-person  = 0.15 (DS = 2.61), (Cl= -0.53, 0.83), P = 0.66  Total test score: TR vs in-person  = 0.03 (SD = 1.64), (Cl= -0.39, 0.46), P = 0.87 |  |
| Niemeijer, Van Waelvelde & Smits-Engelsman (2015) | MABC-2 | 1172 children  within each age band:  AB1 = 431 children  AB2 = 333 AB3 = 408  (No further information) | Cross-cultural validity between Dutch and UK children | Independent t-tests | Aiming and Catching 1:  Age band 1 – t= -0.22, p=.82  Age band 2 – t=4.40, p <.001  Age band 3 – t= 5.20, p<.001  Aiming and Catching 1 (other hand)  Age band 3 – t= 5.30 p<.001  Aiming and Catching 2  Age band 1 – t= -0.85 p=.40  Age band 2 – t= 0.94 p= .35  Age band 3 – t= -0.25, p= .81  Balance 1  Age band 1 – t= 1.38 p=.17  Age band 2 – t=3.70, p <.001  Age band 3 – t=6.12, p<.001  Balance 1 (other leg)  Age band 1 – t= 0.74, p= .46  Age band 2 – t= 4.65, p<.001  Balance 2  Age band 1 – t= -3.06, p= .002  Age band 2 – t= -0.07 p=.95  Age band 3 – t=6.33, p<.001  Balance 3  Age band 1 – t=3.26, p=.001  Age band 2 – t=3.05 p=.002  Age band 3 – t= 1.61, p=.11  Balance 3 (other leg)  Age band 2 – t= 4.22, p<.001  Age band 3 – t=3.19, p=.002  Aiming and Catching Total  Age band 1 – t= -0.72 p=.47  Age band 2- t=3.49, p <.001  Age band 3 – t= 4.64, p<.001  Balance Total  Age band 1 – t=0.68, p=.50  Age band 2 – t=4.88, p<.001  Age band 3 – t=7.55, p<.001  Total test score  Age band 1 – t= -0.91 p=.36  Age band 2 – t= 5.37, p <.001  Age band 3- t=7.04, p <.001 | **61%** |
| Novak et al (2016) | KTK | 2479 children aged between 6-11 years  Females = 1179 and Males = 1300 | Criterion Validity (Concurrent) (KTK 3 and KTK 4) | Pearson correlation | 6 years:  Boys: n = 135, r = 0.96**  Girls: n = 166, r = 0.97**  Total sample: n = 301, r = 0.96**  7 years:  Boys: n = 228, r = 0.97**  Girls: n = 195, r = 0.97**  Total sample: n = 423, r = 0.97**  8 years:  Boys: n = 250, r = 0.98**  Girls: n = 236, r = 0.97**  Total sample: n = 486, r = 0.97**  9 years:  Boys: n = 276, r = 0.97**  Girls: n = 280, r = 0.98**  Total sample: n = 556, r = 0.98**  10 years:  Boys: n = 214, r = 0.97**  Girls: n = 148, r = 0.97**  Total sample: n = 362, r = 0.97**  11 years:  Boys: n = 197, r = 0.98**  Girls n = 154, r = 0.98**  Total sample: n = 351, r = 0.98**  Total:  Boys: n = 1300, r = 0.97**  Girls: n = 1179, r = 0.97**  Total sample: n = 2479, r = 0.97** | **56%** |
|  |  |  |  | Chi-Squared, Cohens Kappa | Chi^2^=6822.53, p<0.001; Kappa = 0.72 |  |
| Okuda, Pangelinan, Capellini & Moreira (2019) | MABC-2 and BOT-2 | BOT-2: 187 elementary school students (grades 1 to 6) (mean age: 113 +- 20 months; boys: n = 117, 62.56%). SLD (n = 20; 10.7%)  MABC-2 : 127 elementary school students (grade 1) (mean age: 76 =- 2 months; boys: n= 58, 45.67%). | Structural validity | Confirmatory factor analysis and bifactor analysis (CFA) | BOT-2  CFA with four dimensions were: χ2(14) = 20.937, p = 0.1135; CFI = 0.988; TLI = 0.976; RMSEA = 0.050 (90% confidence interval [90%CI] = 0.000 to 0.093).  Considering the bifactor model for BOT- 2: χ2(17) = 38.545, p = 0.0021; CFI = 0.962; TLI = 0.938; RMSEA = 0.082 (90%CI = 0.048 to 0.117).  MABC-2 CFA with three dimensions were: χ2(32) = 46.569, p = 0.0463; CFI = 0.92; TLI = 0.89; RMSEA = 0.06 (90%CI = 0.008 to 0.095)  Considering the bifactorial model for MABC-2: χ2(26) = 25.560, p = 0.4875; CFI = 1.000; TLI = 1.004; RMSEA = 0.000 (90%CI = 0.000 to 0.069). | **65%** |
| Psotta & Abdollahipour  (2017) | MABC-2 | Two samples of children:  7- 10-year-olds  (n = 484, 248 boys and 236 girls)  11-16-year-olds (n = 674, 328 boys and  346 girls) | Structural Validity  Hypothesis testing validity | Confirmatory Factor analysis | Age band 2  χ^2^(30)=40.612, p= 094,CMIN/df = 1.354, RMSEA =0.027, GFI = 0.980, AGFI= 0.964,TLI= 0.972  Additional factor loading of Bal 3o MD (-0.27, p<.0001) and MD 3 on AC (-0.28, p=.009)  Age band 3  χ^2=^ 42.081, p=070, CMIN/df= 1.403, RMSEA= 0.024, GFI= 0.984, AGFI= 0.970, and TLI= 0.958. | **69%** |
| Re, Logan, Cattuzzo, Henrique, Tudela, & Stodden, (2018) | TGMD-2 and KTK | 424 healthy children (47%  girls and 53% boys) aged between 5 and 10 years old  Demographics:  White (62%), Black (13%) “Mixed” (25%)  The sample was grouped as follows:  5–6 years (n = 158, 76 girls; M age = 5.78, SD = 0.46 years),  7–8 (n = 204, 98 girls; M  age = 8.03, SD = 0.54 years),  9–10 (n = 62, 27 girls; M  age = 9.56, SD = 0.35 years) | Criterion Validity (Concurrent) | Pearson Correlation | 5–6 years old: r = 0.52, r2 = 0.27  7–8 years old: r = 0.50, r2 = 0.25  9–10 years old: r = 0.34, r2 = 0.12 | **69%** |
|  |  |  |  | Paired sample t-test on percentile ranks | 5–6 years: t= −3.029(157), p= .003  7–8 years: t= −11.134 (203) p <.001  9–10 years: t= −7.243 (61) p<.001  All: t= −11.711 (423), p <.001 |  |
| Rintala, Saakslahti & Livonen (2017) | TGMD-3 | 60 Finnish children (aged 3-9 years old) divided into three separate samples of 20:  Intra-rater reliability study:  Rater A:  Boys n = 10, (ages 6 to 9 years (M = 7.8 ± 1.2)), and Girls n = 10, (ages 5 to 9 years (M = 7.4  ± 1.2))  Rater B:  Boys n = 8, (ages 4 to 7  years (M = 6.6 ± 1.4)), and Girls n = 12, (ages 3 to 7 years (M = 6.1 ± 1.6))  Additional  inter-rater  reliability sample:  Boys n = 10, (ages 4 to 6 years (M = 5.9 ± 0.7)) and Girls n = 10, (ages 5 to 6 years (M = 6.2 ± 0.5)) | Intra-rater reliability | Kappa statistic  Percentage agreement calculation  Intraclass correlation coefficient (with upper and lower boundary) | **Rater A**  Run: κ=0.58  Gallop: κ= 0.8  Hop: κ =0.51  Skip: κ =0.75  Horizontal jump: κ = 0.61  Slide: κ =0.58  Two hand strike on a stationary ball: κ =0.84  One hand force and strike on self-bounced ball: κ =0.70  One hand stationary dribble: κ =0.67  Two hand catch: κ = 0.90  Kick a ball stationary: κ = 0.62  Overhand throw: κ =0.84  Underhand throw: κ =0.85  Locomotor skills: κ =0.69  Ball skills: κ =0.77  Total skills: κ =0.75  **Rater B**  Run: κ =0.42  Gallop: κ =0.77  Hop: κ =0.62  Skip: κ =0.86  Horizontal jump: κ =0.68  Slide: κ = 0.61  Two hand strike on a stationary ball: κ =0.47  One hand force and strike on self-bounced ball: κ =0.73  One hand stationary dribble: κ =0.72  Two hand catch: κ =0.81  Kick a ball stationary: κ= 0.76  Overhand throw: κ =0.68  Underhand throw: κ =0.84K  Locomotor skills: κ =0.73  Ball skills: κ =0.73  Total skills: κ =0.73 | **54%** |
|  |  |  | Inter-rater reliability | Kappa, ICC | Run: κ =0.63 ICC= 0.63  Gallop: κ =0.62, ICC=0.61  Hop: κ =0.19 ICC=0.13  Skip: κ =0.87 ICC=0.87  Horizontal jump: κ =0.38 ICC=0.37  Slide: κ =0.45 ICC=0.45  Two hand strike on a stationary ball: κ =0.32K, ICC=0.32  One hand force and strike on self-bounced ball: κ =0.64 ICC=0.64  One hand stationary dribble: κ =0.81 ICC=0.81  Two hand catch: κ =0.84 ICC=0.84  Kick a ball stationary: κ =0.52 ICC=0.50  Overhand throw: κ =0.65 ICC=0.65  Underhand throw: κ =0.63 ICC=0.62  Locomotor skills: κ =0.57 ICC=0.56  Ball skills: κ =0.64 ICC=0.64  Total skills: κ =0.62 ICC=0.62 |  |
| Rosblad & Gard (1998) | MABC | 60 children (Females = 28  and Males = 32).  Ages ranging from 73 to 83  months (mean = 66 months)  None of the children had any known disabilities. | Cross Cultural Validity (Sweden and America) | T-test | Ball skills:  Catching bean bag (no. out of 10) P = 0.857  Rolling ball into goal (no. out of 10) P = 0.002  Static and dynamic balance:  One-leg balance, preferred leg(s) P = 0.225  One-leg balance, non-preferred leg (s) P = 0.017  One-leg balance, right leg (s) P = 0.102  One-leg balance, left leg (s) P = 0.040  Jumping over cord (no. of trials to pass) P = 0.052  Walking heels raised (no. of steps) P = 0.861 | **39%** |
| Rudd et al (2016) | TGMD-2  and KTK | In total 158 children aged 6-12 years old. (M age = 9.5 SD 2.2)  Females = 72  Males = 86 | Structural validity KTK  Hypothesis testing validity | Confirmatory Factor Analysis | Adequate model fit:  χ2 (2df) = 1.49, P = .47, χ2/df = 0.75, CFI = 1.00, SRMR = .01, RMSEA = .01, P CLOSE = .60 | **43%** |
|  |  |  | Structural validity for the TGMD  Hypothesis testing validity | Confirmatory Factor Analysis | Locomotor:  χ2 (9df) = 9.21; P = .42; χ2/df = 1.02; CFI = .99; SRMR = .05; RMSEA = .01;  PCLOSE = .69    Object control:  χ2 (9) = 27.54; χ2/df = 1.34; P = .001; CFI = .80; SRMR = .07; RMSEA = .11; PCLOSE = .02  This original model was inadequate, so it was revised:  χ2 (8) = 10.13, P = .26; χ2/df = 1.26; CFI = .98; SRMR = .04; RMSEA = .04;  PCLOSE = .52    FMS hierarchical model:  χ2(52) = 71.07; P = .04; χ2/df = 1.36; CFI = .86; SRMR= .07; RMSEA = .05; PCLOSE = .52  The effect of object control on overall fundamental movement skill = .67  The effect of locomotor on overall fundamental movement skill = .39 |  |
|  |  |  | Inter-rater reliability | Bland-Altmann | Locomotor - 95% limit -0.7 to 0.7  Object control skills 95% limit -0.6 to 0.6.  95% confidence within one 1SD (1.96) and contains zero |  |
| Ruiz et al (2003) | MABC | Spanish study:  Total n = 385  (Females = 183  Males = 202)  Japanese study:  Total n = 102  Females = 49  Males = 53  American Study:  Total n = 521  Females = 284  Males = 237 | Cross Cultural Validity | MANOVA | Age band 2  F= 25.07(16), p=.000  Age band 3  F= 35.73(16), p=.000 | **25%** |
| Schulz et al (2011) | MABC 2 Test | 1172 children , aged 3–16 years.  (Females = 606  Males = 566)  AB1 n = 431  AB2 n = 333  AB3 n = 408 | Structural validity | Confirmatory factor analysis | Age band 1:  3 correlated factors was rejected x2 (df = 32) = 410.65, p < 0.001 RMSEA = 0.17, NNFI = 0.76, AGFI = 0.70, SRMR = 0.19  3 factor plus general factor: x2(df = 24) = 33.44, p < 0.095, RMSEA = 0.03, NNFI = 0.99, AGFI = 0.96,  SRMR = 0.023  Age band 2:  3 correlated factors was rejected x2 (df = 32) = 124.6, p < 0.001, RMSEA = 0.094, NNFI = 0.83, AGFI = 0.85, SRMR = 0.089  Double loadings for balance: x2 (df = 27) = 37.70, p = 0.08; RMSEA = 0.035, NNFI = 0.98, AGFI = 0.95, SRMR = 0.038  Age band 3  3 correlated factors was rejected x2 (df = 32) = 71.05, p < 0.001 RMSEA = 0.055, NNFI = 0.93, AGFI = 0.93, and SRMR = 0.056  Double loadings: x2 (df = 28) = 38.41, p = 0.09,  RMSEA = 0.030, NNFI = 0.98, AGFI = 0.96, and SRMR = 0.036 | **62%** |
| Simons et al (2008) | TGMD-2 | In total 99 children aged 7-10 years with cognitive delay.  Mean age = 8 years, 10 months (SD = 1 year, 9 months)  Females = 32  Mean age = 8 years, 8 months (SD = 10 months)  Males = 67  Mean age = 9 years, 8 months (SD = 1 year, 2 months) | Structural validity  Hypothesis testing validity | Confirmatory factor analysis | chi-square = 83.772, DF = 53, p = 0.004, GFI =.88, AFGI = .82. | **61%** |
|  |  |  | Internal consistency | Cronbach’s alpha | Locomotor α = .82  Object control α = .86  Gross Motor Quotient α = .90 |  |
|  |  |  | Test-retest | Spearman correlation | Locomotor = .90  Object Control = .92  GMQ = .98 |  |
|  |  |  | Inter-rater reliability | Pearson correlation | Locomotor = 1.00; p < .05  Object Control = 1.00; p < .05  GMQ = 1.00; p < .05 |  |
| Smits-Engelsman, Fiers, & Henderson (2008) | MABC (Dutch translation) | In total 9 children with  movement difficulties  (Females = 3,  Males = 6)  Ages ranged from 4 to 12 years of age | Inter-rater reliability | Kappa | Average = .99 | **50%** |
| Spironello, Hay, Missiuna, Faught, & Cairney (2010) | BOT (short form) and MABC | 2278 children aged from 9 to 10 years old  From the total sample, the study sample  assessed on BOT-SF and M-ABC:  n = 340 | Criterion Validity (Concurrent) | Pearson correlation | r = .50, P < 0.01 | **93%** |
|  |  |  |  | KAPPA (relative Improvement Over Chance) | 5^th^ percentile:  κ = .19  RIOC = 29.41%  15^th^ percentile:  κ = .29  RIOC = 46.8% |  |
| Stearns, Wohlers, McHugh, Kuzik, & Spence  (2019) | PLAYbasic and CAMSA | In total 102 children  October 2014:  N = 54  Mean age (SD) = 11.10 (1.36)  Age Range = 8.98 to 13.85  Female = 28 (52%)  Male = 26 (48%)  March 2015:  N = 48  Mean age (SD) = 11.48 (1.31)  Age Range = 9.27 to 14.12  Female = 21 (44%)  Male = 27 (56%) | Inter rater reliability (for PLAYbasic and PLAYfun) | Intraclass correlation | PLAYbasic  October 2014:  Average measures =.84**, 95%CI = .73, 9.1  Single Measures = .72**, 95%CI= .57, .83    March 2015:  Average measures =.88**, 95%CI = .79, 9.4  Single Measures = .79**, 95%CI= .65, .88  PLAYfun  October 2014  Average measures = .88***, 95%CI = .79,93  Single measures = .78***, 95%CI = .65,86    March 2015  Average measures = .90***, 95%CI = .82, .94  Single measures = .82***, 95%CI = .70, .89 | **43%** |
|  |  |  | Internal consistency (for PLAYbasic and PLAYfun) | Cronbach's alpha | PLAYbasic  October 2014:  Mean between raters = .61  Rater 1 = alpha .62  Rater 2 = alpha .65    March 2015:  Mean between raters = .6  Rater 1 = .61  Rater 2 = .56  PLAYfun  October 2014:  Mean between raters = .87  Rater 1 = alpha .87  Rater 2 = alpha .86    March 2015:  Mean between raters = .87  Rater 1 = alpha .87  Rater 2 = alpha .83 |  |
|  |  |  | Criterion Validity (Concurrent)  Hypothesis testing validity | Pearson correlation | Between PLAYbasic and CAMSA  October 2014:  Mean = .48**  Rater 1 = .47**  Rater 2 = .41**    March 2015:  Mean = .51**  Rater 1 = .40**  Rater 2 = .61**  Between PLAYfun and CAMSA  October 2014:  Mean = .51**  Rater 1 = .47**  Rater 2 = .50**    March 2015:  Mean = .58**  Rater 1 = .51**  Rater 2 = .60** |  |
| Tan, Parker & Larkin (2001) | BOT-SF and MABC | In total 69 children  (Females = 25,  Males = 44)  between the  ages 4 years, 8 months to 10 years, 8 months (M = 81.8 months, SD = 19.4 months) | Criterion Validity (Concurrent) | Spearman rank | *r* = .79 | **57%** |
| Utesch et al (2016) | MOT 4-6 | 1467 children (aged between 3-6 years)  Girls = 672 (45.8%)  Boys = 795 (54.2%) | Structural validity | Rasch partial credit model | First-step analysis (all items of MOT 4-6) =  *CR* =.032, *pCR* = .43; *P- χ²* = -.356, *pP- χ²* = .55  Follow-up model (global model fit with ordered threshold  Parameters) = (*CR* = .1.964, *pCR* = .06; *P- χ²* = -.227, *pP- χ²* = .24, *RA* = .79) – five items removed | **79%** |
|  |  |  |  | Mixed Rasch model | *CR* = 23, *pCR* = .28; *P- χ²* = -.53, *pP- χ²* = .7,  *RA_class 1* = .63; RA_class 2 = .45 |  |
| Valentini (2012) | TGMD-2 | In total 2,674 children  (Females = 1322, Males = 1352 boys)  Ages ranged from 3 to 10 years old  (M age = 7.56 years, SD = 1.91 years) | Content validity | Content validity index | Clarity CVI = .93  Pertinence CVI = .91 | **6**1% |
|  |  |  |  | Exploratory factor analysis | RMSEA = .06, 90% CL [.06, .07]  CFI (.88), NFI (.09), TLI (.83), GFI (.98), and AGFI (.95) |  |
|  |  |  | Test-retest reliability | Pearson correlation | Overall test: r = .9, p = .001  Locomotor subtest: r = .83, p = .0001  Object control subtest: r = .91, p = .0001  Run: r = .8, p = .001  Gallop: r = .51, p = .001  Hop: r = .57, p = .001  Leap: r = .54, p = .001  Horizontal jump: r = .76, p = .001  Slide: r = .71, p = .001  Striking stationary ball: r = .66, p = .001  Stationary dribble: r = .9, p = .001  Catch: r = .64, p = .001  Kick: r = .9, p = .001  Overhand throw: r = .72, p = .001  Underhand throw: r = .92, p = .001 |  |
|  |  |  |  | T-test | Overall test: t=.9, p=.37  Locomotor subtest: t=.23, p=.82  Object control subtest: t=1.61, p=.11  Run: t=1.68, p=.09  Gallop: t=.73 p=.46  Hop: t=.98, p=.33  Leap: t=.33, p=.74  Horizontal jump: t=1.49, p=.14  Slide: t=1.65, p=1  Striking stationary ball: t=.5, p=.61  Stationary dribble: t=1.78, p=.08  Catch: t=.42, p=.68  Kick: t=2, p=.06  Overhand throw: t=.28, p=.78  Underhand throw: t=1.55, p=.12 |  |
|  |  |  | Criterion Validity (Concurrent) | Pearson correlation | Percentiles for the total sample:  r = .27, p < .001  The correlation explained 7.29% of the variance    Percentiles for each age group:  Age 4: *r* = .42, *p* = .05  Age 5: *r* = .56, *p* = .002  The associations explained 17.6% and 31.4% of the variance, respectively.  Ages 6 to 10: r = .14–.30, p > .05  Children scored significantly higher on the MABC (M percentile=23.57; SD=24.57) compared  with the TGMD-2 (M percentile = 7.50; SD = 10.23). |  |
|  |  |  |  | T-test | Total sample: t(161) = −8.52, p < .001    All age groups (p< .007) |  |
|  |  |  | Intra rater reliability | Cronbach’s alpha | alpha = .92–.99 |  |
|  |  |  | Inter rater reliability | Intraclass correlation | Locomotor subtest = .88  Object control subtest = .89    Locomotor skills = .86–.94  Object control skills = .87–.92 |  |
| Valentini, Ramalho, & Oliveira  (2014) | MABC-2 (Portuguese translation) | In total 844 children (Females = 404  Males = 440)  Aged between 3 and 13 years of age (M = 8.31, SD = 2.91)  Demographics: 59.6% = White 40.4% = Non-white | Content Validity | Content validity index (%) | Clarity:  Experts 1,2&3 = 71.8  Experts 1&2 = 93.9  Experts 1&3 = 78.9  Experts 2&3 = 74.3    Pertinence:  Experts 1,2&3 = 99.2  Experts 1&2 = 99  Experts 1&3 = 99.3  Experts 2&3 = 98.5 | **50%** |
|  |  |  |  | Kappa | Clarity:  Experts 1&2 (IC 95%) = .88 (.76-.99), p<.001  Experts 1&3 (IC 95%) = .80 (.65-.95), p=.001  Experts 2&3 (IC 95%) = .76 (.59-.93), p=.001    Pertinence:  Experts 1&2 (IC 95%) = .92 (.83-.90), p<.001  Experts 1&3 (IC 95%) = .83 (.69-.98), p=.001  Experts 2&3 (IC 95%) = .87 (.59-.93), p<.001 |  |
|  |  |  | Inter-rater reliability | Intraclass correlation | Manual dexterity:  Raters A&B = .99  Raters A&C = .99  Raters B&C = .99  Raters A,B&C = .99    Ball skills:  Raters A&B = .92  Raters A&C = .86  Raters B&C = .87  Raters A,B&C = .91    Balance:  Raters A&B = .99  Raters A&C = .93  Raters B&C = .88  Raters A,B&C = .95    MABC-2 score:  Raters A&B = .99  Raters A&C = .96  Raters B&C = .97  Raters A,B&C = .98 |  |
|  |  |  | Intra-rater Reliability | Intraclass correlation | Manual dexterity: Rater Ax2 = .81    Ball skills: Rater Ax2 = .71    Balance: Rater Ax2 = .72    MABC-2 score: Rater Ax2 = .88 |  |
|  |  |  | Structural Validity | Cronbach’s alpha | Overall of the 3 subscales = .78  Manual Dexterity = .77  Ball skills = .52  Balance = .77 |  |
|  |  |  | Criterion validity (predictive) | ANOVA | Significant differences among children identified with DCD, at risk for DCD and TD children (F (2,841) = 722.07, p < .0001, h2 = .63).  Scores of TD children were significantly higher (p-values < .0001)  Scores of children classified as at risk were significantly higher compared to the children with DCD (p-values < .0001). |  |
|  |  |  |  | ICC | ICC = .88; p < .007 |  |
|  |  |  | Criterion Validity (Concurrent) | Pearson’s correlation | TGMD-2 and MABC-2 standards scores =.30, p < .02    In each classification group:  DCD = .54, p = .08  At risk for DCD = .26, p = .20  TD =.05, p = .40 |  |
|  |  |  |  | Dependent t-tests | Children in general group: t (42) = 1.36, p < .18  Children within each classification group (p values range from .16 to .31) |  |
| Valentini, Zanella, & Webster (2017) | TGMD-3 (Brazilian translation) | In total 597 children aged 3 to 10  Females = 302  (age: M = 6.58, SD = 2.06)  Males = 295  (age: M = 6.76, SD = 2.11) | Content validity | Content validity index (%) | Clarity:  Experts 1,2&3 =78  Experts 1&2 = 97  Experts 1&3 = 77  Experts 2&3 = 75    Pertinence:  Experts 1,2&3 = 99  Experts 1&2 = 100  Experts 1&3 = 99  Experts 2&3 = 98 | **60%** |
|  |  |  |  | KAPPA concordance coefficient | Clarity:  Experts 1&2 (IC 95%) = .91 (.88-1), p<.001  Experts 1&3 (IC 95%) = .79 (.62-.96), p=.001  Experts 2&3 (IC 95%) = .77 (.60-.94), p=.001    Pertinence:  Experts 1&2 (IC 95%) = .97 (.88-1), p<.001  Experts 1&3 (IC 95%) = .86 (.72-.99), p=.001  Experts 2&3 (IC 95%) = .86 (.72-.99), p<.001 |  |
|  |  |  | Inter-rater reliability | Intraclass correlation | TGMD-3 Total = .98  Locomotion = .95  Run = .85  Gallop = .91  Hop = .86  Skip = .99  Jump = .89  Slide = .93  Ball Skills = .97  Strike 1 hand = .96  Strike 2 hands = .94  Dribble = .97  Catch = .96  Kick = .86  Overhand throw = .96  Underhand throw = .97 |  |
|  |  |  | Intra rater reliability | Intraclass correlation | TGMD-3 Total = .90  Locomotion = .90  Run = .61  Gallop = .71  Hop = .86  Skip = .81  Jump = .73  Slide = .78  Ball Skills = .85  Strike 1 hand = .73  Strike 2 hands = .68  Dribble = .90  Catch = .90  Kick = .69  Overhand throw = .60  Underhand throw = .72 |  |
|  |  |  | Test-retest reliability | Pearson correlation | TGMD-3 Total = .90  Locomotion = .93  Run = .60  Gallop = .71  Hop = .82  Skip = .74  Jump = .67  Slide = .74  Ball Skills = .81  Strike 1 hand = .73  Strike 2 hands = .72  Dribble = .73  Catch = .86  Kick = .73  Overhand throw = .71  Underhand throw = .77 |  |
|  |  |  | Internal consistency | Cronbach’s alpha | TGMD-3-BR (α) = .74  Locomotion skills (α) = .63  Ball skills (α) = .76    Skill-to-test and -subtests by sex:  Boys (α) = .76, α values .72 to .76  Girls (α) = .74, α values .71 to .74    Subtests independently:  Boys:  Locomotion skills = .62, α values .59 to .62  Ball skills = .76, α values .72 to .76  Girls:  Locomotion skills = .64, α values .61 to .64  Ball skills = .71, α values .68 to .71    Performance-criteria-to-test and –subtest:  TGMD-3 = .93  Locomotion skills = .90  Ball skills = .88    Performance-criteria-to-test and -subtests by sex:  TGMD-3-BR for boys = .93, α values .90 to .92  TGMD-3-BR for girls = .92, α values .92 to .92  Subtest independently  Boys:  Locomotion skills = .89, α values .87 to .89  Ball skills = .87, α values .85 to .87  Girls:  Locomotion skills = .91, α values .89 to .91  Ball skills = .85, α values .83 to .85 |  |
|  |  |  | Structural validity | Confirmatory factor analysis | Factor loading:  Locomotion:  Run = .46  Gallop = .41  Hop = .56  Skip = .44  Leap = no value  Horizontal jump = .5  Slide = .6  Ball Skills:  Strike 1 hand = .42  Strike 2 hands = .63  Dribble = .72  Catch = .58  Kick = .58  Overhand throw = .51  Underhand throw = .55    Run- SE=.03, skills-subtest correlation =.5**  Gallop SE=.069, skills-subtest correlation =.62**  Hop- SE=.053, skills-subtest correlation =.66**  Skip- SE=.056, skills-subtest correlation =.62**  Leap – no values  Horizontal jump - SE=.045, skills-subtest correlation =.55**  Slide – SE- NO VALUE, skills-subtest correlation =.73*  Strike 1 hand - SE=.128, skills-subtest correlation =.6**  Strike 2 hand- SE=.129, skills-subtest correlation =.7**  Dribble - SE=.198, skills-subtest correlation =.76**  Catch- SE=.103, skills-subtest correlation =.62**  Kick- SE=.108, skills-subtest correlation =.64**  Overhead throw - SE=NO VALUE, skills-subtest correlation =.63**  Underhand throw - SE=.105, skills-subtest correlation =.6** |  |
| Valentini, Rudisill, Bandeira, & Hastie (2018) | TGMD-2 | In total 2,463 children aged between 3 and 10 year olds (M = 8.10, SD = 1.32)  Females= 1344  Males= 1119 | Structural validity | CFA | Run *h*(communalities=.52), LOC=.54  Gallop *h*(communalities=.72), LOC=.83  Hop *h*(communalities=.57), LOC=.69  Strike *h*(communalities=.57), OC=.75  Kick *h*(communalities=.58), OC=.76  Throw *h*(communalities=.57), OC=.73  Two factors explained 59.33% of the variance (locomotor factor: 19.56%; object control factor: 39.76%).    RMSEA (0.06, 90% confidence interval [0.06, 0.07]; CFI: 0.94; NFI: 0.94; TLI: 0.83; GFI: 0.98; and AGFI: 0.95 | **61%** |
|  |  |  | Internal consistency | Cronbach’s alpha | Locomotion = .60  Run =.46  Gallop = .54  Hop =.37  Object control = .66  Strike = .54  Kick = .59  Throw = .55  General test = 0.70 |  |
|  |  |  |  | Bivariate correlation  (between activity and subtest) | Run = .64, p=<.001  Gallop = .59, p<.001  Hop = .7, p=<.001  Strike = .68, p<.001  Kick = .68, p<.001  Throw = .74, p<.001 |  |
|  |  |  |  | Intraclass correlation | Locomotion = .67  Object control = .68  SF of TGMD = .71 |  |
|  |  |  | Inter-rater reliability | Intraclass correlation | Locomotion:  A&B = .94, A&C = .91, B&C = .92, A&B&C = .94    Run:  A&B = .87, A&C = .81, B&C = .82, A&B&C = .87    Gallop:  A&B = .94, A&C = .89, B&C = .83, A&B&C = .90    Hop:  A&B = .92, A&C = .93, B&C = .92, A&B&C = .93    Object Control:  A&B = .96, A&C = .93, B&C = .94, A&B&C = .96  Strike:  A&B = .89, A&C = .83, B&C = .84, A&B&C = .89    Kick:  A&B = .96, A&C = .90, B&C = .85, A&B&C = .92    Throw:  A&B = .96, A&C = .95, B&C = .94, A&B&C = .95 |  |
|  |  |  | Intra rater reliability | Intraclass correlation | Locomotion:  A = .95, B = .96, C = .94    Run:  A = .94, B = .97, C = .95    Gallop:  A = .96, B = .97, C = .93    Hop:  A = .96, B = .95, C = .95    Object Control:  A = .97, B = .98, C = .96    Strike:  A = .97, B = .99, C = .95  Kick:  A = .96, B = .97, C = .96    Throw:  A = .95, B = .96, C = .94 |  |
|  |  |  | Test-retest reliability | Correlation analysis  (not specified) | Locomotor r = .87  Run r = .84  Gallop r = .55  Hop r = .61  Object control r = .95  Strike r = .7  Kick r = .94  Throw r = .76 |  |
| Valentini et al. (2015) | TGMD-2  MABC | 424 children (220 boys and 204 girls, age range: 4–10 years)  DCD = 58  At risk of DCD = 133  TD = 233 | Criterion Validity (Concurrent) | Pearson correlation | TGMD-2 Locomotor and MABC Ball Skills =.202  TGMD-2 Locomotor and MABC Balance =.187  TGMD-2 Locomotor and MABC Total =.169  TGMD-2 Object control and MABC Ball Skills =.289  TGMD-2 Object control and MABC balance =.207  TGMD-2 Object control and MABC Total =.316  TGMD-2 Total and MABC Ball Skills =.244  TGMD-2 Total and MABC Balance =.181  TGMD-2 Total and MABC Total =.226 | 33% |
|  |  |  | Inter-rater Reliability (TGMD-2) | Pearson correlation | TGMD-2 locomotor r ranged from .88–.96; object control .89–.94  MABC manual dexterity: r = .96; ball skills: r = .94; balance: r = .97 |  |
| Valtr & Psotta (2019) | MABC-2 | 120 Czech participants of three age groups (17:0 – 17:11 years: months, 18:0-18:11 years: months, 19:0-19:11 years: months), n= 40 (20 boys, 20 girls) in each age group. | Structural Validity | Confirmatory factor analysis (CFA) | Age band 3 χ2(9) = 14.035, *p*= .121, CMIN/*df*= 1.559, RMSEA = .069, GFI = 0.966, AGFI = 0.920, and TLI = 0.954.  All factor loadings on the MD or AC latent factor were statistically significant (p < .05). | **54%** |
| Van Waelvelde, De Weerdt, De Cock, & Smits-Engelsman, (2004) | MABC (Dutch version) and tests of ball catching and balance | Sample n = 90 children (50 = boys and 40 = girls)  Control n =  43 children (29 = boys and 14 = girls)  The age groups are as follows:  7–8 years:  N = 107, (71  from the sample group and 36 from the control group). Mean age = 8 years 6 months.  9–10 years:  N = 26, (19 from the sample group and 7 from the control group).  Mean age = 9 years 3 months. | Criterion Validity (Concurrent)  (MABC & Ball catching) | Spearman correlation | 7-8 years: Total impairment score = -.72, p<.01  Speed of one hand = -.51, p<.01  Bimanual coordination = -.45, p<.01  Pen control= -40, p<.01  Ball skills sub score = -.72, p<.01  Catching = -.74, p<.01  Throwing = -.58, p<.01  Balance sub score = -.46, p<.01  Standing on one leg = -.48, p<.01  Jumping = -.19  Balance in walking = -.21    9 years: Total impairment score = -.68, p<.01  Speed of one hand = -.30  Bimanual coordination = -.35  Pen control = -.60, p<.01  Ball skills sub score = -.53, p<.01  Catching = -.54, p<.01  Throwing = -.27  Balance sub score = -.48, p<.01  Standing on one leg = -.45, p<.01  Jumping = -.18  Balance in walking = -.51, p<.01 | **66%** |
|  |  |  | Criterion Validity (Concurrent)  (MABC & KTK jump) | Spearman correlation | 7-8 years: Total impairment score = -.76, p<.01  Speed of one hand = -.54, p<.01  Bimanual coordination = .47, p<.01  Pen control= -.52, p<.01  Ball skills sub score = -.5, p<.015  Catching = -.57, p<.01  Throwing = -.44, p<.01  Balance sub score = -.70, p<.01  Standing on one leg = -.65, p<.01  Jumping = -.41, p<.01  Balance in walking = -.37, p<.01    9 years: Total impairment score = -.69, p<.01  Speed of one hand = -.43, p<.05  Bimanual coordination = -.39, p<.05  Pen control = -.47, p<.05  Ball skills sub score = -.58, p<.01  Catching = -.44, p<.01  Throwing = -.49, p<.01  Balance sub score = -.65, p<.01  Standing on one leg = -.48, p<.05  Jumping = -.33  Balance in walking = -.58, p<.01 |  |
|  |  |  | Criterion Validity (Concurrent)  (MABC & KTK beam) | Spearman correlation | 7-8 years: Total impairment score = -.72, p<.01  Speed of one hand = -.57, p<.01  Bimanual coordination = .43, p<.01  Pen control= -.46, p<.01  Ball skills sub score = -.52, p<.01  Catching = -.53, p<.01  Throwing = -.38, p<.01  Balance sub score = -.68, p<.01  Standing on one leg = -.63, p<.01  Jumping = -.30, p<.01  Balance in walking = -.46, p<.01    9 years: Total impairment score = -.58, p<.01  Speed of one hand = -.37  Bimanual coordination = -.19  Pen control= -.20  Ball skills sub score = -.34  Catching = -.34  Throwing = -.26  Balance sub score =-.69, p<.01  Standing on one leg = -.66, p<.01  Jumping = -.50, p<.01  Balance in walking = -.38 |  |
| Wagner, Webster & Ulrich  (2017) | TGMD-3 (German translation) | In total 189 typically developing children (Females = 90,  Males = 99)  Mean age = 7.15 years (SD = ± 2.02 years Age Range = 3.17-10.67 years  56 = kindergarten children  133 = elementary school children | Test-retest reliability  Hypothesis testing validity | Intraclass correlation | Locomotor skills = .94, 95% CI [.91, .96], p < .001  Balls skills = .98, 95% CI [.97, .99], p < .001 | **70%** |
|  |  |  | Inter-rater reliability  Hypothesis testing validity | Intraclass correlation | Locomotor skills = .88, 95% CI [.76, .95], *p* < .001  Ball skills = .97, 95% CI [.94, .99], *p* < .001 |  |
|  |  |  | Intra-rater reliability  Hypothesis testing validity | Intraclass correlation | Locomotor skills = .97, 95% CI [.94, .99], *p* < .001  Ball skills = .99, 95% CI [.98, 1.00], *p* < .001 |  |
|  |  |  | Internal consistency  Hypothesis testing validity | Cronbach’s alpha | Locomotor skills = .76  Ball skills = .89 |  |
|  |  |  | Structural validity  Hypothesis testing validity | Confirmatory factor analysis | Locomotion:  Run: IR = .32  Gallop: IR = .17  One legged hop: IR = .47  Skip: IR =.42  Horizontal jump: IR = .37  Slide: IR = .47    Ball skills:  One hand forehand strike: IR = .69  One hand stationary dribble: IR = .63  Two hand catch: IR = .44  Kick a stationary ball: IR = .63  Overhand throw: IR = .63  Underhand throw: IR = .52    Divergent measures:  Locomotor: FR=.77, AVE=.38, FLR = 1.77  Ball skills: FR= .90, AVE =.62, FLR = 1.09 |  |
|  |  |  | Criterion Validity (Concurrent)  Hypothesis testing validity | Spearman correlation | Ball skills:  At time of testing = *rs*(89) = .36, *p* < .001  12 months after = *rs*(66) = .39, *p* < .001  Locomotor:  At the time of testing - *rs*(89) = .15, *p* = .086, *1-ß* = .42  12 months after= *rs*(66) = .08, *p* = .253, *1-ß* = .16 |  |
| Ward, Thornton, Lay, Chen & Rosenberg (2020) | TGMD-2 | 16 primary school students (age 8.2 ± 2.2 years)and 17 raters were recruited to the current study; 7 pediatric movement professionals (age 28.7 ± 6.55 years) and 10 primary school teachers (age 34.5 ± 13.5 years). | Inter-rater reliability  Hypothesis testing validity | ICCs and Spearman correlations (Rs) | All assessments Overall ICC (Video): Rater 1 (Pediatric professionals) = .88**(95% CI = 0.80-0.93) Rs = 0.75** Rater 2 (Primary teachers) = .84** (95% CI =0.75–0.90) Rs = .73**  All assessments Overall ICC (Point light): Rater 1 = .87**(95% CI 0.79–0.93) Rs = 0.79** Rater 2 = .85** (95% CI 0.77–0.91) Rs = .67**  Individual Skills (Video): Kick ICC Rater 1 = .92** (95% CI = .80- .98)  Rater 2 = .87** (95% CI = .71- .96)  Throw ICC Rater 1 = .92** (95% CI = .80- .98)   Rater 2 = .89** (95% CI = .74 -.97)  Hop ICC  Rater 1 = .86** (95% CI = .67 - .96)  Rater 2 = .80** (95% CI = .53 - .91)  Jump ICC  Rater 1 = .75** (95% CI = .42 - .93) Rater 2 = .59** (95% CI = .05 - .88)  Individual Skills (Point light): Kick ICC  Rater 1 = .92** (95% CI = .80- .98) Rater 2 = .86** (95% CI = .69- .96)  Throw ICC Rater 1 = .94** (95% CI = .86- .98) Rater 2 = .90** (95% CI = .78 -.97)  Hop ICC Rater 1 = .74** (95% CI = .39 - .93) Rater 2 = .72** (95% CI = .35 - .92)  Jump ICC Rater 1 = .86** (95% CI = .68 - .96) Rater 2 = .71** (95% CI = .34 - .92) | **43%** |
| Wagner, Kastner, Petermann, & Bos (2011) | MABC-2 | In total 323 children  (Female = 154,  Male = 169)  Mean age of 8.96 years  (min: 7.02, max: 10.98). | Structural validity | Confirmatory factor analysis | Aiming and catching: Factor reliability = .43 and average assessed variance = .28  Throw tennis: Indicator reliability = .15  Throw beanbag: Indicator reliability = .44, t(factor loading) = 2.89  Balance: Factor reliability = .53 and average assessed variance = .45  One foot: Indicator reliability = .46  WAL: Indicator reliability = .29, t(factor loading) = 6.04  Hop: Indicator reliability = .08, t(factor loading) = 4  Divergent measures:  Average assessed variance:  Manual dexterity = .44  Aiming and catching = .28  Balance = .45  Maximum squared intercorrelation:  MD & BL = .55  BL & AC = .28  Fornell-Larcker Ratio:  MD = 1.25  AC = 1.01  BL = 1.22 | **62%** |
| Wilson, Kaplan, Crawford, & Dewey (2000) | BOT-LF | In total 50 children aged between 7 years, 1 month and 14 years, 5 months (M = 10.34 years, SD = 1.83).  Male = 33  Female = 17  26 children had known learning or attentional problems (LD) or both.  24 children did not have any known learning problems. | Inter-rater reliability | Intraclass correlation | Entire sample:  Battery composite ICC = .945  Gross motor composite ICC = .897  LD:  Battery composite ICC = .939  Gross motor composite ICC = .898  Non LD:  Battery composite ICC = .892  Gross motor composite ICC = .853  DCD:  Battery composite ICC = .939  Gross motor composite ICC = .816  Non DCD:  Battery composite ICC = .934  Gross motor composite ICC = .902  Running speed and agility = .902  Balance = .817  Bilateral motor coordination = .93  Strength = .84  Upper Limb coordination = .825 | **53%** |
|  |  |  |  | KAPPA | Battery composite = .64  Gross motor composite = .7 |  |
| Wuang & Su (2009) | BOT-2 | Final sample (n = 100). Female = 41 and Male = 59  Average age = 82.9 months S.D. = 24.9, Age Range = 48–124 months.  64 children =  mild ID  36 children = moderate  to severe ID | Internal consistency | Cronbach’s alpha | Upper limb coordination = .87  Bilateral coordination = .87  Balance = .85  Running speed and agility = .87  Strength = .85  Manual coordination = .88  Body coordination = .87  Strength and agility = .88  TOTAL = .920 | **57%** |
|  |  |  | Test-retest reliability | Intraclass correlation (with a 2-way random effects model) | Upper limb coordination (95%CI) = .88 (.83 - .92)  Bilateral coordination (95%CI) = .96 (.95 - .98)  Balance (95%CI) = .99 (.98 - .99)  Running speed and agility (95%CI) = .97 (.95 - .97)  Strength (95%CI) = .96 (.95 - .97)  Manual coordination (95%CI) = .98 (.97 - .99)  Body coordination (95%CI) = .99 (.98 - .99)  Strength and agility (95%CI) = .99 (.97 - .99)  TOTAL (95%CI) = .99 (.99 - 1) |  |
|  |  |  |  | Standard error of measurement | Upper limb coordination = .73  Bilateral coordination = .65  Balance = .49  Running speed and agility = .49  Strength = .63  Manual coordination = .66  Body coordination = .8  Strength and agility = .8  TOTAL = 1.79 |  |
| Wuang, Su & Su  (2012) | MABC-2 | The final sample (n=144)  Females = 57  Males = 87  Mean age = 7 years 7 months (SD 2y 1mo,  range 6y–12y 9mo) | Internal consistency | Cronbach’s alpha | Aiming and Catching = .84  Balance subscales = .88  MABC-2 Test total score: a = .90 | **51%** |
|  |  |  | Test-retest reliability | Intraclass correlation (with a two-way random effects model) | AC1 = .88, 95%CI = .83 - .92, SEM = .74  AC2 = .96, 95%CI = .95 - .98, SEM = .61  AC OVERALL = .91, 95%CI = .82 - .95, SEM = .92    BL1 = .99, 95%CI = .98 - .99, SEM = .35  BL2 = .97, 95%CI = .95 - .98, SEM = .44  BL 3 = .96, 95%CI = .95 - .97, SEM = .62  BL TOTAL = .97, 95%CI = .95 - .98, SEM = .52    TOTAL SCORE = .97, 95%CI = .96 - .98, SEM = .52 |  |
| Wuang, Lin & Su (2009) | BOT-2 | 446 children with intellectual deficits aged 4-18 years  Female = 40.4%  Male = 59.6%  Mean age was 9.4 years (S.D. = 4.02)  71.7% of children = classified as having mild ID  28.3% of children = classified as having moderate to severe ID | Structural validity | Rasch analysis (partial credit model) (IRT) | Original BOT  18/53 misfitting items  Manual coordination PSI = 4.14 (0.95)  Body Coordination PSI = 2.02 (0.80)  Strength and Agility PSI = 4.24 (0.95)  BLC4 and BAL 5 had disordered thresholds  14 items had disordered step difficulty, so items were re-scored  Revised BOT  No misfitting items  99.8% of the variance accounted for  No DIF for age or gender  Unidimensional underlying construct | **69%** |
| Zhu et al (2011) | PE Metrics | 5021 students  Male = 2568 (51.1%)  Female = 2453 (48.9%)  The sample was split into:  K = 1465,  G2 = 1991  G5 = 1565 | Structural validity | Many-faceted rasch model (IRT)  Grade 2 analysed first - K and G5 anchored onto G2 scale | All Infit and Outfit statistics of G2, K and G5 within acceptable “–2 to 2” range  Acceptable age progression | **43%** |
| Zoia et al (2018) | MABC-2 (Italian translation)   \|  \| \| --- \| | IT sample:  AB1:  338 children between the ages of 3 and 6 years  162 females and 176 males  AB2:  380 children between the  ages of 7 and 10 years  199 females and 181 males  UK sample:  AB1:  431 children, aged 3 to 6 years  AB2:  333 children aged 7 to 10 years | Structural Validity | Confirmatory Factor analysis | AGE BAND 1:  Satorra-Bentler X2 (df = 23) = 57.42, p < .01, RMSEA = .067 (p = .096), NNFI = .96, AGFI = .93, SRMR = .054  All model parameters significant (t-value > 1.96)  AGE BAND 2:  Satorra-Bentler X2 (df = 30) = 78.46, p < .01, RMSEA = .065 (p = .073), NNFI= .95, AGFI = .92, SRMR = .067  All model parameters significant (t-value > 1.96) | **46%** |
|  |  |  | Cross cultural validity (Italy and UK) | ANOVA  (Bonferroni and LSD adjustment) | Country effect 11/27 raw scores (p < .01), ES low to moderate (ηp2: .014 - .09)  Age Band 1  AC1 in 3-4 years (F(1,414) = 9.536; p = .002), interaction effect (F(1,414) = 4.103; p = .043): at 3 years of age, IT children made fewer catches (F(1,414) = 10.985, p = .001; Cohen’s d: .48)  Dynamic BAL1 (F(1,762) = 42.76; p < .001) IT children made less correct steps  Age band 2  AC1 (F(1,319) = 31,659; p < .001): IT children achieved a higher number of correct catches  Static BAL both legs (best leg: F(1,705) = 13,581; p < .001; other leg: F(1,705) = 21,675; p < .001),  IT children maintained balance longer  Dynamic BAL1 (F(1,705) = 32,423; p < .001), Age X Country (F(3,705) = 4.270; p = .005): better result for UK children  Dynamic BAL2 ‘other leg’ (F(1,705) =  12,768; p < .001), IT children made a higher number of correct hops |  |
| Zuvela, Bozanic, & Miletic (2011) | FMS POLYGON  TGMD-2 | 95 children (48 boys and 47 girls) aged 8 years old (8.1 ± 0.3) | Intra-rater reliability | Intraclass correlation | Tossing and catching a volleyball against the wall consecutively = .92  Running across obstacles = .96  Carrying the medicine balls = .90  Straight running = .95    Overall test = .98 | **42%** |
|  |  |  | Structural validity | Factor analysis | Tossing and catching a volleyball against the wall consecutively: mean = 5.57, SD = 1.24, F = .84  Running across obstacles: mean = 4.91, SD = .59, F = .87  Carrying the medicine balls: mean = 5.33, SD = .59, F = .86  Straight running: mean = 4.53, SD = .31, F = .83 |  |
|  |  |  | Criterion Validity (Concurrent)  Hypothesis testing validity | Pearson's r correlation | r= -.82, p < .05 |  |
