# Supplementary material for: The validity and reliability of observational assessment tools available to measure fundamental movement skills in school-age children: A systematic review
Source: PLoS One. 2020 Aug 25;15(8):e0237919. doi: 10.1371/journal.pone.0237919 (PMC7447071; doi:10.1371/journal.pone.0237919)
Supplement: Attachment — Submitted filename: POLOS.docx [file pone.0237919.s004.docx]

This article focuses on a very important topic and is of acceptable quality. But to improve the article, there are the following suggestions:

1- What is the main problem of the research statement? Why are researchers focused on observational tools? This is not in the introduction.
2- Search terms are very limited. There are many articles or tools that do not have the term "fundamental or skills" in their titles. This strategy may limit the number of attained articles.
3- Quality evaluation of selective researches is required. This index can show the quality of any research and illustrate the accuracy and dependability of findings. There are many ways and means to evaluate the quality of research.
4- Data extraction criteria have been taken arbitrarily. Using the standard method for evaluation and review can be understandable and useful. The Consensus-Based Standards for the Selection of Health Status Measurement Instruments (COSMIN) is an internationally accepted manner to measurements assessment. Thus this is suggested that the results section of the article arrange according to the COSMIN checklist. This can easily and clearly illustrate the pros and cons of each measurement. This matter also can show the pitfalls and disadvantages of tools collectively. It is not only beneficial in the selection of instruments for clinical practice but also shows issues that are necessary to be considered in new studies for revising and development of new measures.
5- The vast amount of discussion - especially in the beginning - is the paragraph of the result. Results ought to be discussed, not to repeat.
6- Fortunately, in the final sections of the discussion, the authors return to the discussion, and from a broad perspective, they talk about the shortcomings and opportunities of the tools and offer useful and consciousness-raising advice to clinicians and researchers. I believe that this approach should be the dominant frame of discussion and that authors should integrate the findings with theoretical literature, and finally guide clinics, schools, and institutions to gain insight into the problems in this area.

Kind Regards

Saeed
